# Generation and maturation of human iPSC-derived 3D organotypic cardiac microtissues in long-term culture

**DOI:** 10.1038/s41598-022-22225-w

**Published:** 2022-10-18

**Authors:** Ece Ergir, Jorge Oliver-De La Cruz, Soraia Fernandes, Marco Cassani, Francesco Niro, Daniel Pereira-Sousa, Jan Vrbský, Vladimír Vinarský, Ana Rubina Perestrelo, Doriana Debellis, Natália Vadovičová, Stjepan Uldrijan, Francesca Cavalieri, Stefania Pagliari, Heinz Redl, Peter Ertl, Giancarlo Forte

**Affiliations:** 1 Center for Translational Medicine (CTM), International Clinical Research Centre (FNUSA-ICRC), St. Anne’s University Hospital, Studentská 812/6, 62500 Brno, Czech Republic; 2 Faculty of Technical Chemistry, Institute of Applied Synthetic Chemistry and Institute of Chemical Technologies and Analytics, Vienna University of Technology, 1040 Vienna, Austria; 3 Faculty of Medicine, Department of Biomedical Sciences, Masaryk University, 62500 Brno, Czech Republic; 4 Electron Microscopy Facility, Fondazione Istituto Italiano Di Tecnologia, Via Morego 30, 16163 Genoa, Italy; 5 Department of Chemical Engineering, The University of Melbourne, Parkville, VIC 3010 Australia; 6 Dipartimento di Scienze e Tecnologie Chimiche, Università degli Studi di Roma Tor Vergata, via della Ricerca Scientifica 1, 00133 Rome, Italy; 7 Ludwig Boltzmann Institute for Experimental and Clinical Traumatology, AUVA Research Center, 1200 Vienna, Austria; 8 Austrian Cluster for Tissue Regeneration, 1200 Vienna, Austria; 9 Department of Biomaterials Science, Institute of Dentistry, University of Turku, 20014 Turku, Finland

**Keywords:** Cardiovascular models, Heart development

## Abstract

Cardiovascular diseases remain the leading cause of death worldwide; hence there is an increasing focus on developing physiologically relevant in vitro cardiovascular tissue models suitable for studying personalized medicine and pre-clinical tests. Despite recent advances, models that reproduce both tissue complexity and maturation are still limited. We have established a scaffold-free protocol to generate multicellular, beating human cardiac microtissues in vitro from hiPSCs—namely human organotypic cardiac microtissues (hOCMTs)—that show some degree of self-organization and can be cultured for long term. This is achieved by the differentiation of hiPSC in 2D monolayer culture towards cardiovascular lineage, followed by further aggregation on low-attachment culture dishes in 3D. The generated hOCMTs contain multiple cell types that physiologically compose the heart and beat without external stimuli for more than 100 days. We have shown that 3D hOCMTs display improved cardiac specification, survival and metabolic maturation as compared to standard monolayer cardiac differentiation. We also confirmed the functionality of hOCMTs by their response to cardioactive drugs in long-term culture. Furthermore, we demonstrated that they could be used to study chemotherapy-induced cardiotoxicity. Due to showing a tendency for self-organization, cellular heterogeneity, and functionality in our 3D microtissues over extended culture time, we could also confirm these constructs as human cardiac organoids (hCOs). This study could help to develop more physiologically-relevant cardiac tissue models, and represent a powerful platform for future translational research in cardiovascular biology.

## Introduction

Cardiovascular diseases (CVD) remain the leading cause of death worldwide^1–4^, and developing new therapies is still a major challenge, since a significant number of drug candidates fail to pass clinical trials, or are withdrawn from the market due to adverse side effects^5–7^. In order to approve safer and more effective therapies, there is an increasing demand to develop faithful models of human heart tissue for pre-clinical research^7^. While recent technologies provide some insight into how human CVDs can be modelled in vitro, a comprehensive overview of the complexity of the human heart remains elusive due to the limited cellular heterogeneity, physiological complexity, or maturity of the constructs produced^8^. Furthermore, animal models may not always faithfully reflect the unique features of human biology and disease, and could give rise to ethical concerns^9–11^.

Induced pluripotent stem cell (iPSC) technology has revolutionized the differentiation and derivation of cardiomyocytes for personalized disease modelling and drug testing^8,12–20^. However, when cultured in 2D as models for development, disease and toxicology^18,21,22^, cardiomyocytes do not reflect the 3D complexity of the native tissue, where the geometry, the presence of different cell types and their interaction with the extracellular matrix (ECM) play a crucial role.

Early 3D cardiac tissue models called “cardiospheres” were developed by culturing human heart tissue biopsies, or by mixing non-isogenic populations of cardiomyocytes, non-myocytes and biocompatible hydrogels^23–25^; however, such microtissues generally have limited culture continuity, self-organization and fail to capture the heterogeneity characteristic of organotypic models. Recently, cardiac tissue engineering technologies have enabled the development of more physiologically-relevant tissue models, which entail a higher degree of complexity, organization and dynamics^26–29^, such as engineered heart tissues (EHTs)^27,30,31^, isogenic cardiac microtissues^24,32–37^, and organs-on-a-chip^38–48^.

Organoids, defined as 3D miniaturized versions of an organ, are emerging as promising tools showing realistic micro-anatomy, and organ specific function^9,49–51^. In order to be considered as an "organoid", an in vitro model must fulfil specific requirements, including: (1) 3D multicellular composition with organ-specific cell types, (2) self-organization and histological resemblance to the tissue of origin and (3) recapitulation of at least one specialized biological function similar to the organ being modelled^51–53^.

Well-established organoids have been already generated for the brain, kidneys, intestines, guts, lungs and many other organs^9,54^, while organoid models of the heart have only started to emerge in the last couple of years^55,56^. Notably, early mammalian cardiac organoids showing spontaneous self-organization with distinct atrium- and ventricle- like regions were generated from mouse pluripotent stem cells (PSCs)^57,58^, or as a part of gastruloids^59^. Shortly afterwards, human PSC-derived cardiac organoid models were described^56,60–65^, which were developed with different approaches varying from assembling different cardiac cell types^62^, followed by other self-organized models more faithful to cardiac-specific development^56,63–66^.

Recently reported human cardiac organoids include single chamber models—namely the left ventricle in the case of “cardioids”^56^—, relying on an external ECM scaffold, such as Matrigel^61,63^, display chamber-like structures^56,64^, featuring the co-emergence of gut tissue together with atrial- and ventricular-like regions^63,65^. While being extremely informative, most of these models are generated by short-term culture, while long-term culture could help them acquire a more mature phenotype, a feature which is usually desirable for more physiologically relevant in vitro tissue models^67,68^.

Here, we aimed to establish a simple protocol to generate induced pluripotent stem cell (iPSC)-based human 3D cardiac microtissues that could be generated in scaffold-free conditions, cultured for extended periods, featuring multiple cell types of the human heart with spontaneous proto-tissue organization, preserve coordinated contractile activity for several months, and functionally responsive to cardioactive drugs—which are hereby referred as 3D human organotypic cardiac microtissues (hOCMTs). By combining RNA-sequencing, ultrastructural and metabolic analyses, we demonstrated that dimensionality and time in culture are crucial mediators of survival, differentiation, collective organization and maturation of hOCMTs. Finally, we confirmed our human organotypic cardiac microtissues (hOCMTs) qualify as 3D human cardiac organoids (hCOs), hence as an in vitro heart model, by proving their response to cardioactive and cardiotoxic drugs in long-term culture.

## Results

### 2D-to-3D culture switch promotes spontaneous cardiac microtissue formation in scaffold-free conditions and proto-tissue organization in long-term culture

Since the 2D monolayer differentiation of induced pluripotent stem cell (iPSC)-derived cardiomyocytes is well established in the literature, and long-term cultures of iPSC-derived cardiomyocyte monolayers naturally tend to delaminate into beating clusters^69^, we first performed human iPSC (hereafter hiPSC) differentiation in a 2D system and tested whether they could be used to generate 3D cardiac aggregates in the absence of any external ECM scaffold.

**Figure 1:**
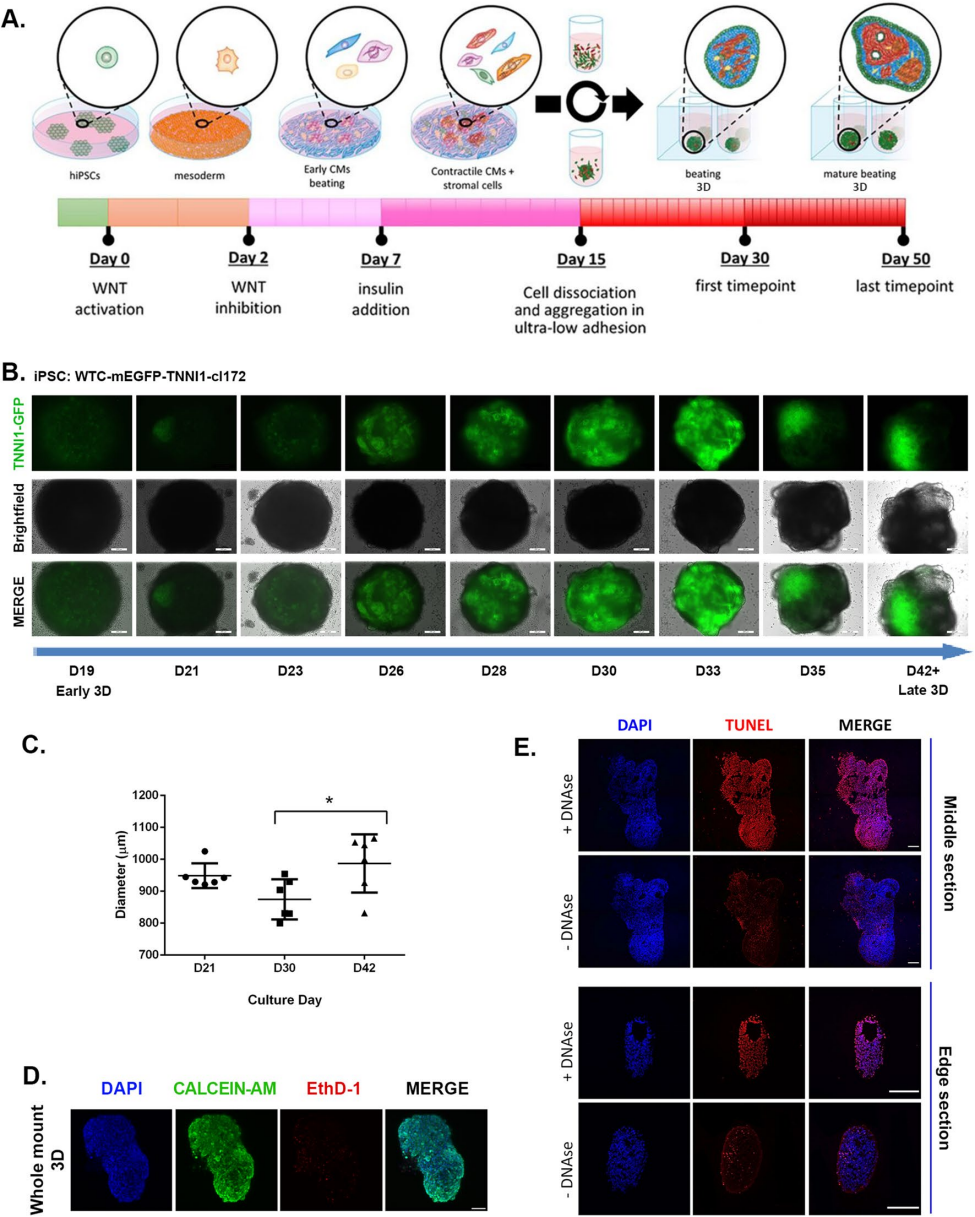
Long-term human cardiac microtissues can be spontaneously generated by scaffold-free conditions in 3D. (**A**) Schematic workflow for the generation and long-term culture of hiPSC derived cardiac microtissues. Warm colours represent cardiomyocyte lineages, while the rest of the colours represent non-myocytes. (**B**) Monitoring spontaneous self-organization of hiPSC-derived cardiomyocytes within 3D microtissues expressing GFP-tagged Cardiac Troponin I reporter over culture time (scale bar = 200 μm). (**C**) Average diameter of 3D long-term cultured cardiac microtissues over time (n = 6, Mean ± SD, Tukey’s multiple comparisons test). (**D**) Cell viability of 3D long-term cultured microtissues with Calcein/AM staining on day 50, green = live, red = dead, blue = DAPI counterstain (Scale bar = 200 µm). (**E**) TUNEL staining (red) depicting apoptotic cells on day 50 3D long-term cultured microtissue cryosections, and positive controls with DNAse I treatment (edge and center). Nuclei were counterstained with DAPI (blue) (scale bar = 200 µm).

Cardiac differentiation was induced in confluent 2D hiPSC monolayers by sequential modulation of the WNT pathway with small molecules, in the absence of insulin^70^ as previously described by Lian et al.^13,14^: First, mesoderm specification was achieved by transient WNT activation through chemical inhibition of GSK3, followed by cardiac mesoderm differentiation through the inhibition of the WNT palmitoleoyltransferase PORCN^13,14^ (Fig. 1A). When beating cell clusters were observed (day 7), insulin supplement was added to the media, since it was needed for cell survival after early contractile cardiomyocytes emerged during differentiation^70^. On day 15 of differentiation, the monolayer was dissociated into single cells and seeded on round-bottom ultra-low attachment plates in order to induce the spontaneous formation of aggregates in the presence of ROCK inhibitor (Fig. 1A). Throughout the study, we used 2D monolayer cultures as controls.

One day after switching the culture from 2 to 3D, we observed under the light microscope a tendency for the spontaneous formation of 3D microtissues without the need for any ECM scaffold, which started to show spontaneous beating behaviour upon removing the ROCK inhibitor after 48 h. To monitor this spontaneous cellular organization, we repeated the experiment to generate microtissues from hiPSC expressing GFP-tagged cardiac troponin I (TNNI1) reporter. As expected, we observed the emergence of GFP-expressing contractile cardiomyocytes together with non-GFP-tagged non-myocyte cells within the microtissues. With time in culture from day 21 to day 42, the GFP-tagged contractile cardiomyocytes and non-myocytes gradually and spontaneously rearranged within the microtissues from a random distribution to more discrete regions (Fig. 1B, Supplementary Fig. 1A). The diameter of the microtissues reached up to 0.9 ± 0.04 mm on day 21 and 1 ± 0.09 mm on day 42, in the absence of any external ECM supplementation (Fig. 1C, Supplementary Video 1 for day 53). The microtissues went on displaying spontaneous contractile activity in long-term culture and until at least day 100 (Supplementary Video 2 for day 107).

This observation suggested that modifying a well-established 2D protocol^13,14^ into our 3D differentiation protocol could give rise to scaffold-free contractile cardiac microtissues that contain not only cardiomyocytes (GFP-tagged), but also other non-myocyte cell populations (non-GFP tagged), which collectively showed a tendency to be spontaneously redistributed in extended culture times.

A common drawback of 3D cultures is the possibility of apoptotic/necrotic core formation due to poor oxygen and nutrient diffusion towards the core of the construct^71^. In order to rule out that this occurs in our extended 3D cultures (day 50), we performed whole mount cell viability assay and despite some physiological levels of dead cells throughout the microtissues, we demonstrated no accumulation of non-viable cells was visible at the core of the constructs (Fig. 1D). Moreover, we complemented this analysis by performing Terminal deoxynucleotidyl transferase (TdT) dUTP Nick-End Labeling (TUNEL) assay, which detects the presence of apoptotic cells, on cryosections obtained from the edge and the core of the 3D tissues. Here we noticed no accumulation of apoptotic cells at either position in the 3D microtissues (Fig. 1E and Supplementary Fig. 1B). Finally, we confirmed the absence of a necrotic/apoptotic core by ultrastructural analysis of the core of the microtissue by transmission electron microscopy (TEM) (Supplementary Fig. 1C).

Due to the presence of intrinsic and spontaneous signs of proto-self-organization, cellular heterogeneity, and functional beating in our 3D microtissues over extended culture time, we will hereafter refer to these constructs as human organotypic cardiac microtissues (hOCMTs).

### 2D-to-3D culture switch prompts the formation of human iPSC-derived organotypic cardiac microtissues composed of multiple heart cell types

One of the key features of *bona fide* organoids is the presence of different cell types typical of the given organ, as well as their ability to self-organize in microstructures similar to the organ being modelled^51–53^.

**Figure 2:**
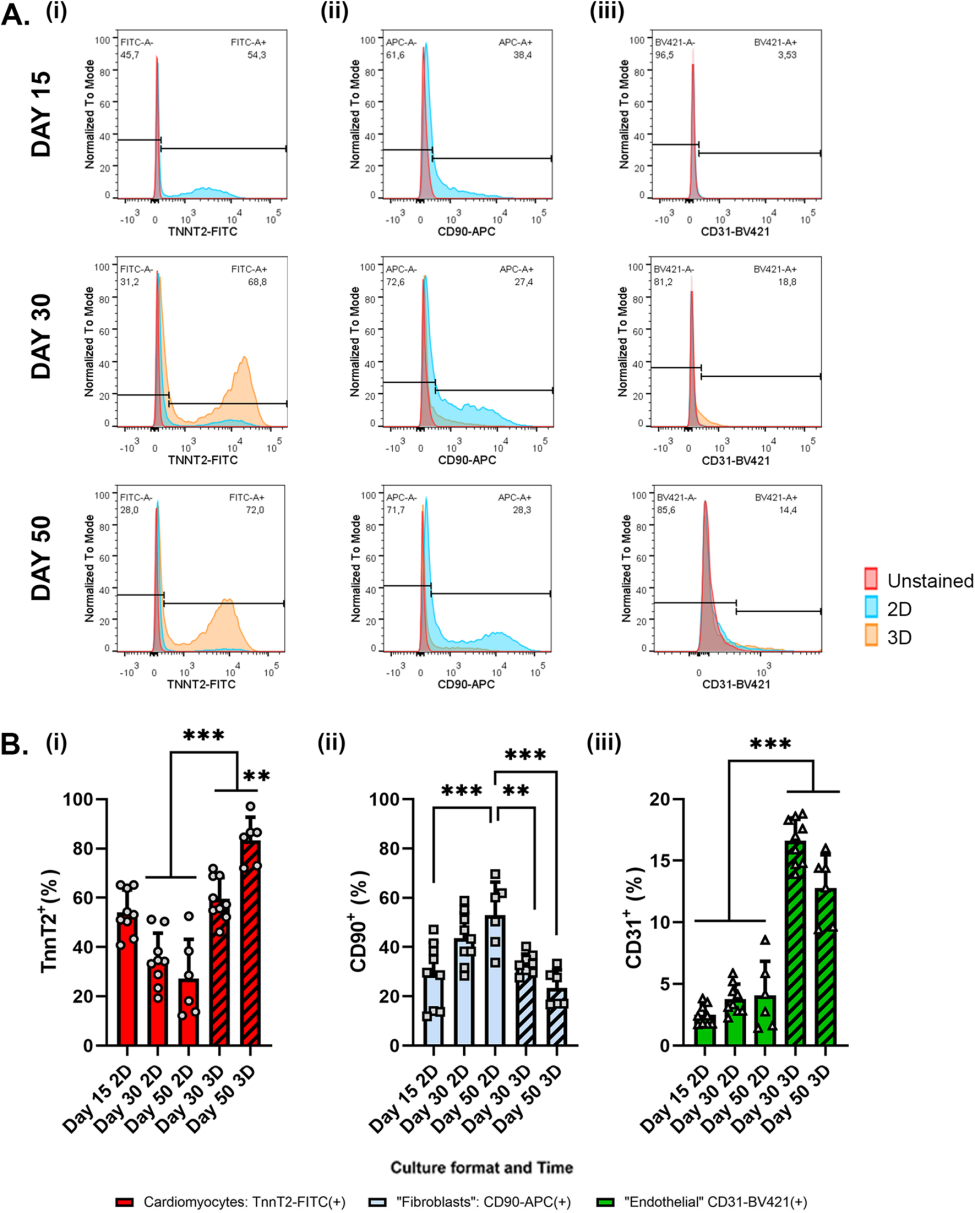
Long-term 3D cultures of hOCMTs contain multiple cardiac cell populations. (**A**) Representative flow cytometry histograms for analysis of cardiac cell types on day 15, day 30 and day 50, with a comparison of 2D and 3D cultures. (i) Cardiomyocytes (TNNT2-FITC), (ii) “Fibroblasts” (CD90-APC), (iii) Endothelial cells (CD31-BV421). Red group represents unstained cells, blue group represents 2D cultures, and orange group represents 3D long-term cultured microtissues, and the gating with the black line depicts the percentage of populations for 3D long-term cultured microtissues. (**B**) Composite average of cardiac cell populations from all flow cytometry experiments: (i) Cardiomyocytes (TNNT2-FITC^+^ %), (ii) “Fibroblasts” (CD90-APC^+^ %), (iii) Endothelial cells (CD31-BV421^+^ %), on day 15, day 30 and day 50, with a comparison of 2D and 3D cultures. The percentage indicates the positive cells versus total cells (mean ± SD obtained in three independent analyses, n = 9 per condition, Tukey's multiple comparisons test).

To assess the cellular heterogeneity of our 3D hOCMTs, we quantified the presence of the three most represented cell populations in the human heart over time^72,73^—cardiomyocytes, fibroblasts and endothelial cells—by flow cytometry at given time-points (day 15, day 30 and day 50, Fig. 2A,B). On day 15 of differentiation (i.e. the first time-point of microtissue aggregation), the flow cytometry analysis of cardiac troponin T2 (TNNT2)-positive population indicated the differentiation into cardiomyocytes in 2D (54.14 ± 9.08%) in addition to a distinct population of CD90-positive non-myocytes (28.87 ± 13.04%). The flow cytometry analysis demonstrated that 3D extended culture was associated with a significant increase in the percentage of TNNT2-positive cardiomyocytes as compared to 2D culture (3D day 50: 83.27 ± 9.45% vs 2D day 50: 27.23 ± 15,86%, p < 0.0001) (Fig. 2B). On the contrary, 2D culture was shown to favour the overgrowth of CD90-positive cells (2D day 50: 52.97 ± 13.44% vs 3D day 50: 23.38 ± 7.35%, p < 0.001) (Fig. 2B). While a very limited number of CD31-positive cells was observed in any of the 2D culture timepoints, a significant increase in cells expressing the endothelial marker CD31 occurred during 3D culture (3D day 50: 12.78 ± 2.71% vs 2D day 50: 4.07 ± 2.76% in, p < 0.001) (Fig. 2B).

These results indicate that 2D-to-3D culture switch promotes the generation of heterogeneous microtissues and favours the survival of the cardiomyocyte population in extended culture.

**Figure 3:**
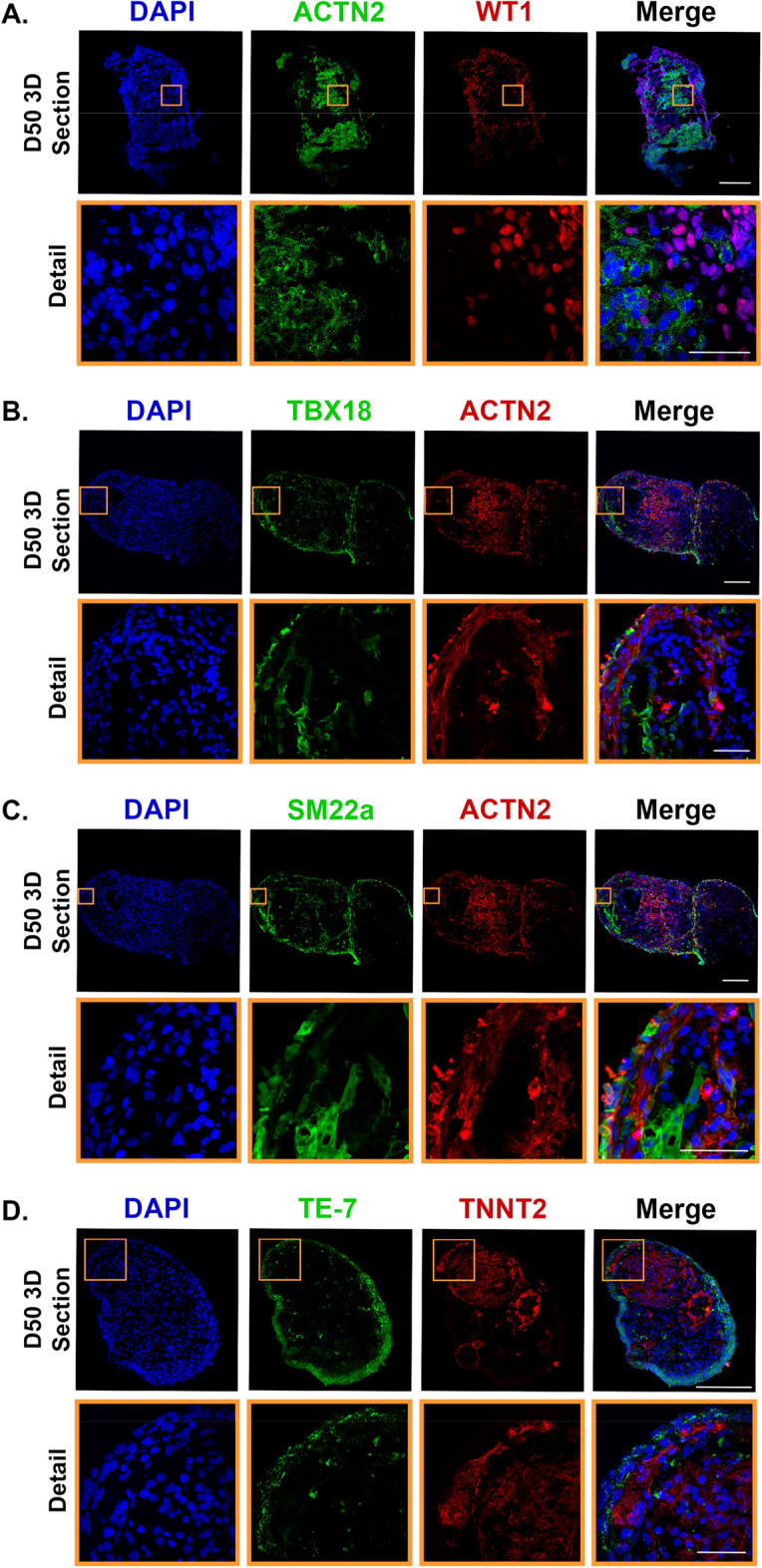
Long-term 3D culture of hOCMTs recapitulates advanced cardiac morphology and cellular heterogeneity in vitro showing markers for cardiomyocyte, epicardial and fibroblast cell types. Immunofluorescence analysis showing the representative co-specification of multiple cell type markers in 3D long-term cultured hOCMT sections on day 50 including cardiomyocytes ([**A**–**D**] ACTN2 in green or red, TNNT2 in red), epicardial cells ([**A**] WT1 in red, [**B**] TBX18 in green, [**C**] SM22a in green), epicardial/fibroblastic and smooth muscle cells ([**C**] SM22a in green), “fibroblasts” ([**D**] TE-7 in green). Nuclei were counterstained with DAPI (blue). Full 3D hOCMTs scale bar = 200 µm, Detail scale bar = 50 µm.

**Figure 4:**
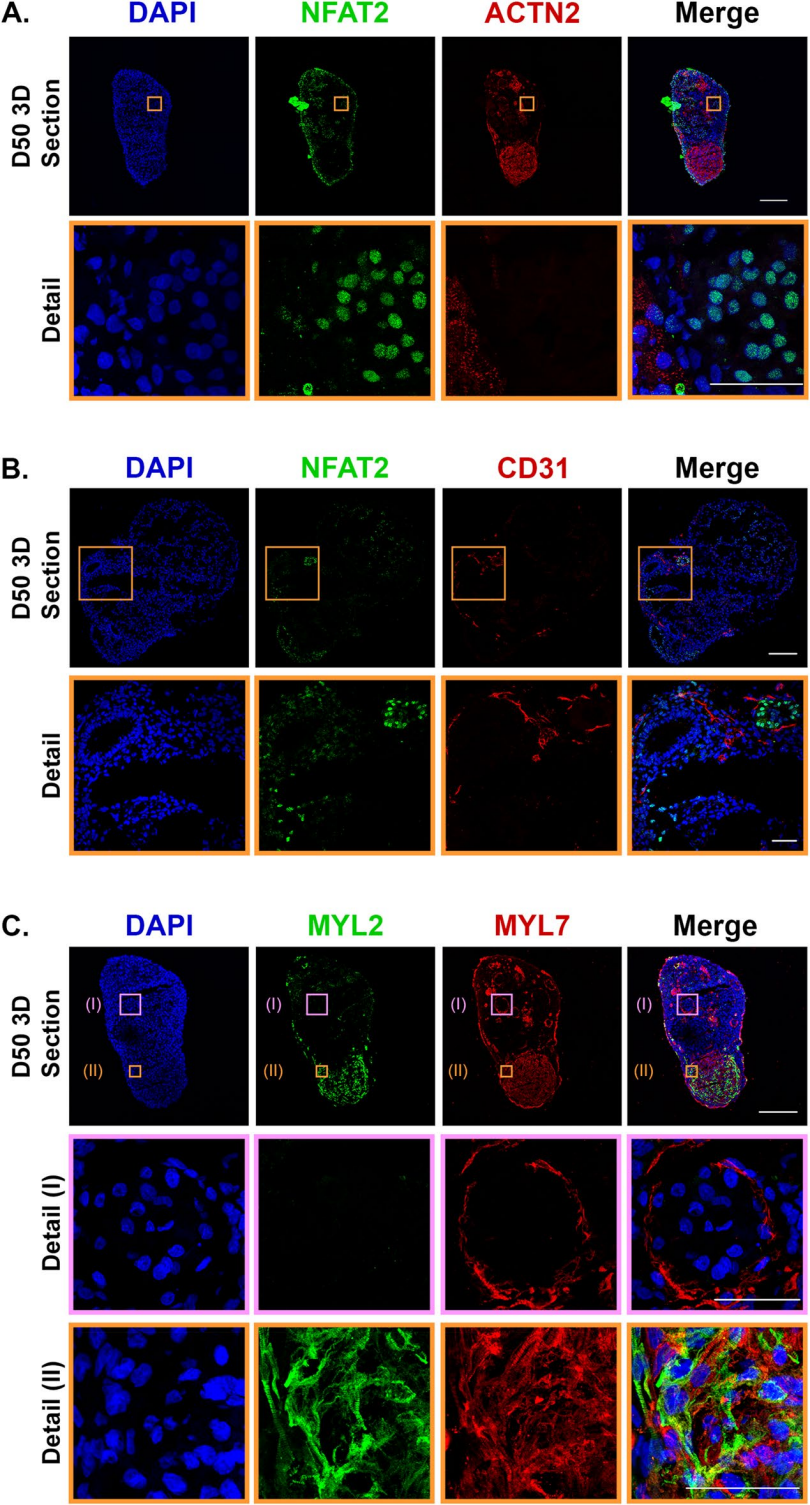
Long-term 3D culture of hOCMTs recapitulates advanced cardiac morphology and cellular heterogeneity in vitro showing markers for endocardial, endothelial, and cardiomyocyte subtypes. Immunofluorescence analysis showing the representative co-specification of multiple cell type markers in 3D hiPSCs-derived cardiac microtissue/hOCMT sections on day 50 including endocardial ([**A**,**B**] NFAT2 in green), endothelial cells ([**B**] CD31 in red), pan-cardiomyocytes ([**A]** ACTN2 in red), atrial ([**C**] MYL7 in green) and ventricular ([**C**] MYL2 in red) cardiomyocyte subtypes. Nuclei were counterstained with DAPI (blue). Full 3D hOCMTs scale bar = 200 µm, Close-up scale bar = 50 µm.

To explore the cellular heterogeneity and the spatial distribution of different cell subsets in our 3D hOCMTs in more detail, cryosections were performed at day 50 of culture and stained to detect the presence of cells expressing markers specific of the different populations in the human heart^73^ (Figs. 3, 4).

Confocal imaging showed that cells located at the periphery of the construct stained positive for markers of epicardial cells Wilms tumor protein 1 (WT1) and TBX18, in addition to epicardial/fibroblast markers TE-7 and SM22a (Tagln), which suggested the presence of these cells primarily forming an outermost layer (Fig. 3A–D). The center of the construct—instead—stained positive for cardiac troponin T2 (TNNT2) and sarcomeric α-actinin (ACTN2), thus describing the formation of a discrete cluster of cardiomyocytes. This result further confirmed the tendency of contractile cells to re-locate to the core of the construct within time in 3D culture that we noticed by using GFP-tagged cardiac troponin I (TNNI1) reporter hiPSC line (see Fig. 1B). Discrete areas could also be stained with endocardial cell markers NFAT2 and endothelial cell marker CD31 (Fig. 4A,B).

Next, we stained for ventricular (MYL2) or atrial (MYL7) Myosin Light Chain isoforms, as specific markers of atrial and ventricular cardiomyocytes or contractile cells at different stages of maturation^74^. Here we could identify two distinct pools of cardiomyocytes that stained either positive for MYL7 only, or co-stained positive MYL2 and MYL7 (Fig. 4C). This result hinted at the possibility that either differentially specified cardiomyocytes or contractile cells at different stages of maturation co-existed with the contractile core of the 3D constructs.

In recent reports, the formation of 3D cardiac organoids was associated with the developmental co-emergence of elements of endoderm and mesoderm tissues resembling the development of the heart and gut in the embryo^63,65^. We thus stained for markers of embryonic (SOX2) or adult gut tissue (ASCL2) together with markers of undifferentiated cells (MESP1, Brachyury) and detected no such cells in day 50 3D constructs (Supplementary Fig. 2A,B).

Furthermore, for cardiac morphogenesis (GATA4), Sarcoplasmic/Endoplasmic Reticulum Calcium ATPase 2 (SERCA2), smooth muscle/fibroblastic cells (α-SMA), and gap junction proteins (Connexin 43, or Cx43) were also observed within the microstructures (Supplementary Fig. 2C).

Altogether, these results confirmed that iPSC-derived 3D hOCMTs favours the survival of cardiomyocytes and contain multiple cell types of the human heart organized in distinct domains.

### Human iPSC-derived organotypic cardiac microtissues display ultrastructural organization and maturation in long-term culture

A key feature of the adult heart is the existence of a highly recognizable three-dimensional ultrastructure due to the periodical repetition of the functional units of the contractile apparatus, the sarcomere. The length and the alignment of the sarcomeres, together with the interspacing of myosin-actin myofilaments and the abundance and shape of mitochondria, are considered representative of the maturity of the contractile tissue.

**Figure 5:**
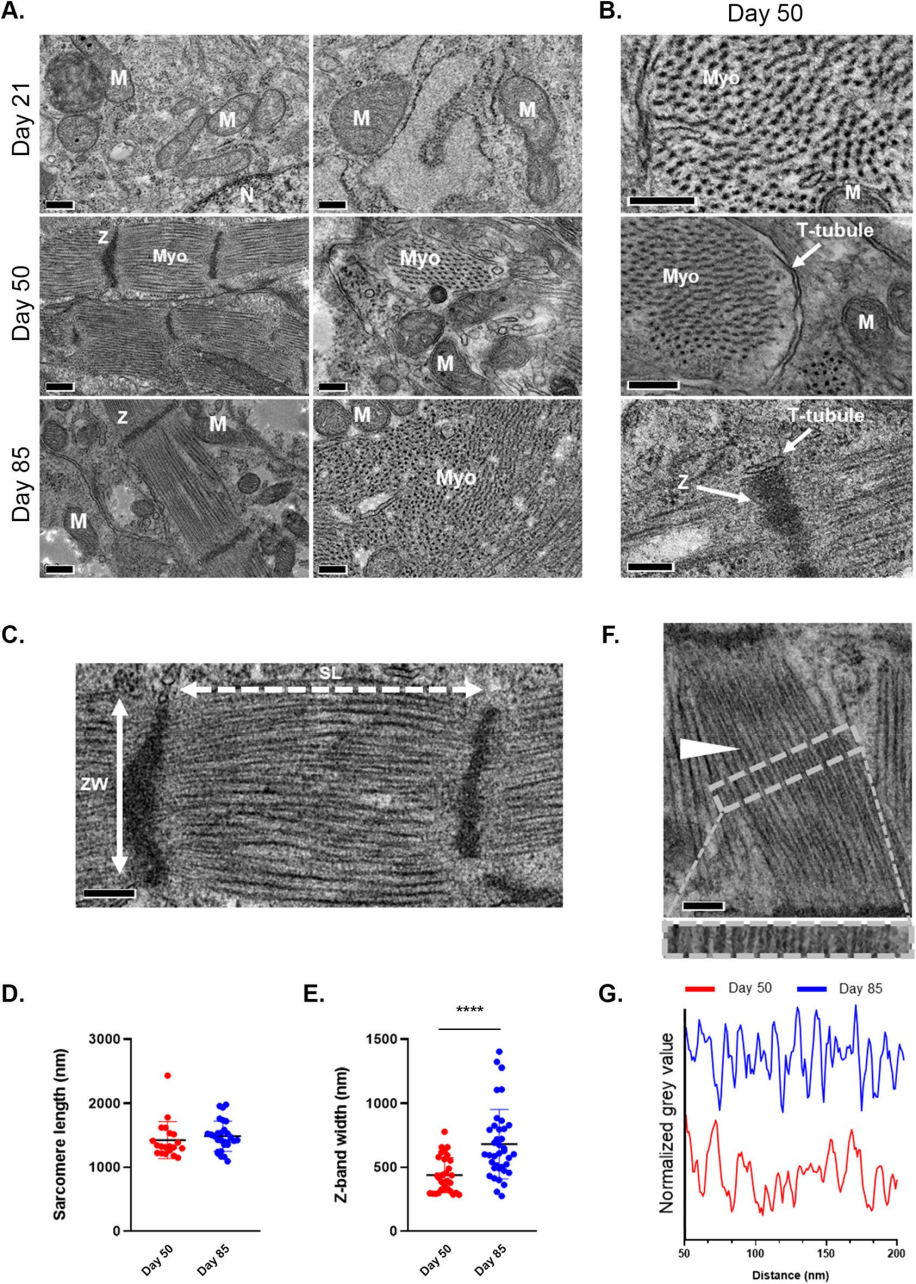
Ultrastructural analysis of 3D hOCMTs indicates enhanced maturation in 3D environment over time. (**A**) TEM images of 3D hOCMTs s showing increasing ultrastructural organization over time, from day 21 to day 85: Cardiomyocyte myofibers (Myo), Mitochondria (M) Z-band (Z), T-tubules. Scale bars = 200 nm. (**B**) Close-up images on day 50. Scale bars = 200 nm. (**C**) Representative micrograph showing sarcomere length (SL; white dotted two-headed arrow) and z-band width (ZW; cross sectional length; white solid two-headed arrow) with the corresponding sarcomere length (**D)**; day 50, 1400 ± 300 nm; day 85, 1500 ± 200 nm; N > 20, Mean ± SD)- and z-band width (**E**); day 50, 450 ± 100 nm; day 85 700 ± 250 nm; N > 30, Mean ± SD). Scale bar = 200 nm. (**F**) Micrograph showing myofiber organization (white arrow) with their relative organization as defined by the plot profile intensity on day 50 vs day 85 (**G**) of the area indicated by the grey dashed box of (**F**). Scale bar = 200 nm.

We analysed the ultrastructure of the contractile core of hOCMTs and assessed how it developed with time in culture by transmission electron microscopy (TEM). TEM analysis clarified that the prototypical contractile apparatus was hardly recognizable in hOCMTs cultured for 21 days, while highly organized sarcomeres with distinct z-disks and evenly distributed myofilaments could be detected in those cultured for 50 days (Fig. 5A,B).

The same analysis also demonstrated that longer culture times (day 85) led to the assembly of t-tubules close to the regularly spaced myofibrils, together with the appearance of packed and elongated mitochondria (Fig. 5F and Supplementary Fig. 3). The presence of t-tubules, extensions of the sarcolemma interspersed around the contractile apparatus to maximize the efficiency of calcium exchange, is only found in mature cardiac muscle and was not reported for standard 2D monolayer cultures^75^. We found the sarcomere length to be approximately 1.5 µm (Fig. 5C,D). This value did not change at later time-points (day 85) and is consistent with the values described for young mammalian cardiomyocytes^67^. Meanwhile, Z-band was found to increase slightly in width, but not significantly, with time in culture (700 ± 250 nm on day 85 vs. 450 ± 100 nm on day 50, p < 0.0001) as to get closer to the values typical of adult heart^67^ (Fig. 5E–G).

Furthermore, TEM also demonstrated a higher amount of mitochondria and glycogen accumulation at later time-points (day 50 and 85) compared to day 21 (Supplementary Fig. 3), which suggests a metabolic shift to advanced maturation of cardiomyocytes^67^. All these features indicate hOCMTs undergo structural and metabolic maturation^67^ when cultured for long time in 3D.

### 3D long-term culture induces human iPSC-derived organotypic cardiac microtissue maturation

Contractile cell maturation can be monitored by tracking the evolution of the expression of specific genes encoding for contractile proteins. Hence, we set at investigating the transcriptional landscape of hOCMTs in order to assess the impact of time and dimensionality on the maturation of the contractile cells.

To this end, we performed bulk RNA-sequencing and differential expression analysis (DE analysis) on day 30 and 50 of the 3D culture and compared them to 2D monolayer cultures harvested at the same time-points (GEO: GSE209997), adult heart tissues and Engineered Heart Tissues (EHTs) obtained from previously published datasets (NCBI Bioproject accession numbers: PRJNA667310^76^ and PRJNA628736^77^ for adult heart, and PRJNA831794^78^ for EHTs [alias GEO: GSE201437]).

**Figure 6:**
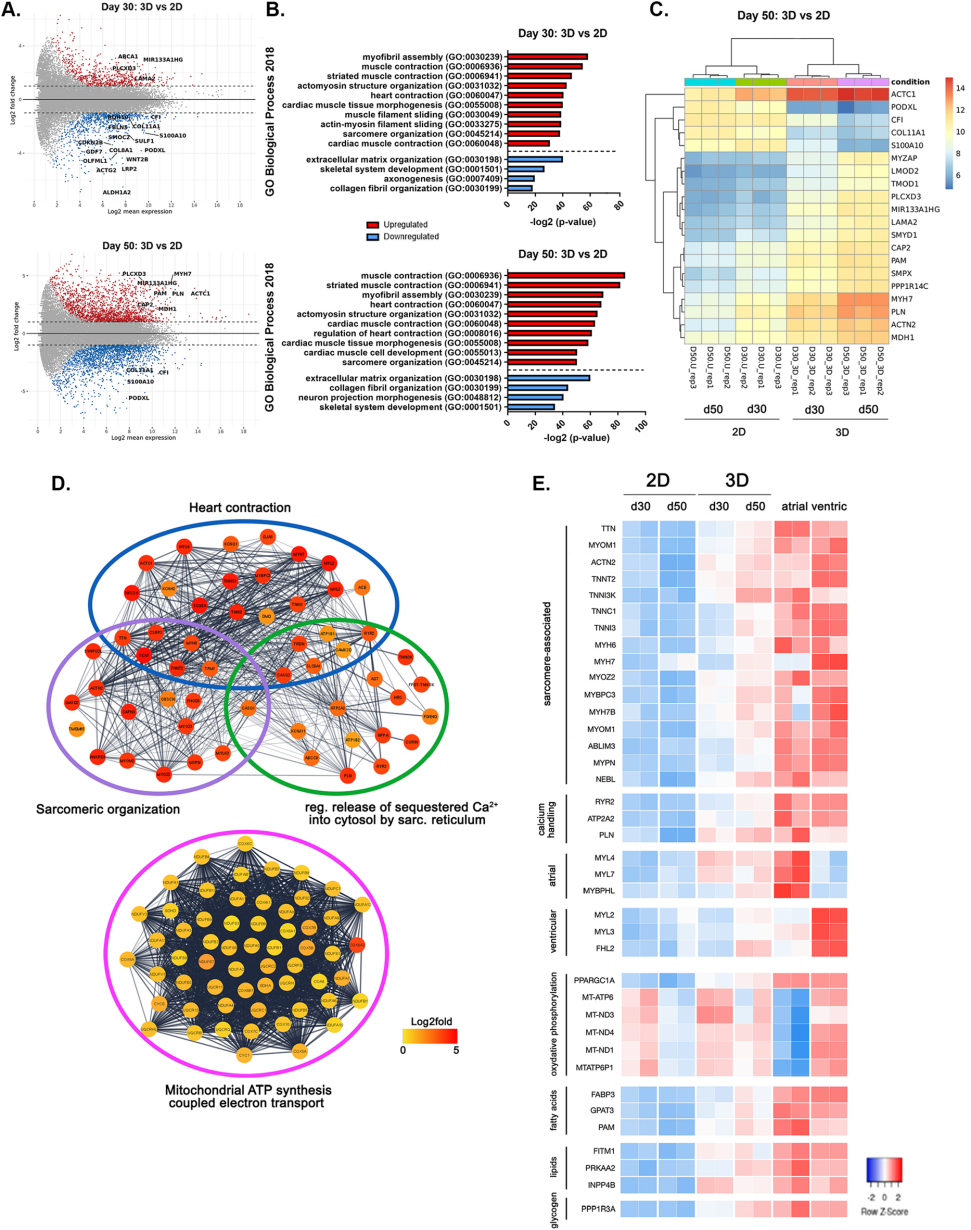
3D long-term cultures induce improved cardiac specification and cardiomyocyte maturation at transcriptional level. (**A**) MA plot of the differentially regulated genes between 2D monolayer and 3D hOCMTs at day 30 (up) and day 50 (down) (log2 mean, p.adj < 0.05). (**B**) Graph representing the − log2value adjusted p-value of significantly upregulated (red) or downregulated (blue) GO Biological process categories when comparing 3D hOCMTs and 2D monolayers at day 30 (up) and day 50 (down) (p.adj < 0.05). (**C**) Heatmap representing the log2fold change for the top 20 differentially regulated genes between 2D monolayer and 3D hOCMTs at day 50 (p.adj < 0.05). (**D**) STRING network of the upregulated genes at day 50 hOCMTs as compared to monolayer cultures for the indicated GO biological processes categories by Cytoscape (Kappa score = 0.3). Log2fold change for the nodes (genes) is represented using a color-coded scale. (**E**) Heatmap encoding for log2fold changes in selected cardiac genes for the 3D hOCMTs and the 2D monolayers at both time-points in comparison with available datasets for adult human atrial and ventricular tissues (p.adj < 0.05).

A total of 2975 genes were found to be significantly and differentially regulated in 3D microtissues compared to monolayer cultures at day 30. This number increased to 6437 at day 50 (Fig. 6A and Supplementary Dataset 1, 2), possibly indicating a bigger divergence between 2 and 3D cultures over time.

The functional annotation at both time-points revealed that the genes differentially regulated 3D hOCMTs had a fingerprint for cardiogenesis, which included the categories of myofibril assembly and sarcomeric organization, muscle contraction and cardiac tissue morphogenesis (Fig. 6B). As an example, the top 20 deregulated genes at day 50 between 3D and monolayer cultures included genes which are well known to be involved in cardiac muscle maturation and function, namely cardiac muscle α-actin (*ACTC1*), sarcomeric α-actinin (*ACTN2*), phospholamban (*PLN*) and myosin heavy chain 7 (*MYH7*) (Fig. 6C). On the contrary, 2D cultures showed an increased expression in ECM-related genes, most likely as a result of the predominance of cardiac fibroblast population in monolayer culture (Fig. 6B,C and Supplementary Fig. 4).

Clustering analysis of the genes upregulated in 3D hOCMTs compared to 2D monolayer cultures at day 50 showed a highly interconnected network of genes involved in heart contraction and sarcomeric organization, which included important structural proteins of the contractile apparatus such as *ACTC1*, *ACTN2*, troponins (*TNNT2*, *TNNC1*, *TNNI1*, *TNNI3*), myosin heavy (*MYH6*, *MYH7*) and light (*MYL2*, *MYL3*) chains, dystrophin (*DMD)*, titin (*TTN)*, obscurin (*OBSCN*) and myozenin 2 (*MYOZ2*), among many others (Fig. 6D).

In good agreement with the presence of t-tubules in 3D hOCMTs and their drift towards a more mature intracellular Ca^2+^-dependent contraction, we detected the enhanced expression of the SERCA Ca-ATPase 2 (*AT2A2*) and its inhibitor phospholamban (*PLN*), triadin (*TRDN*), ryanodine receptors (*RYR2* and the Purkinje’s cell specific *RYR3*), calsequestrins (*CASQ1*, *CASQ2*), as well as genes related to cardio-renal homeostasis angiotensinogen, atrial natriuretic peptide and corin (*AGT*, *NPPA*, *CORIN*). Interestingly, in accordance with the marked increase in the number of mitochondria observed in the TEM images (see Fig. 5A, Supplementary Fig. 3), day 50 3D hOCMTs showed an upregulation in the network of genes associated with mitochondrial ATP synthesis coupled electron transport, including several NADH:ubiquinone oxidoreductase and Cytochrome c oxidase subunits (Fig. 6D).

Since long-term 3D cultures seemed to be able to promote the maturation of hOCMTs, we set at comparing the transcriptomic landscape of our in vitro constructs with available datasets obtained from adult atrial and ventricular heart tissues^76,77^ (Fig. 6E).

Sample clustering showed that day 50 3D hOCMTs were closer to adult heart at transcriptional level, with the most similar levels being associated with the expression of several sarcomere-associated genes, including *TTN*, *ACTN2*, troponins (*TNNT2*, *TNNI3K*, *TTNC1*, *TNNI3*) and Myosin heavy chains (*MYH6, MYH7, MYH7B*), as well as important components of the calcium handling apparatus (*RYR2, ATP2A2, PLN*). Consistent with our immunostaining results, day 50 3D microtissues displayed both atrial (*MYL4*, *MYL7*, *MYBHL*) and ventricular (*MYL2*, *MYL3*, *FHL2*) chamber markers (Fig. 6E). In accordance with the increased number of mitochondria (see Fig. 5A, Supplementary Fig. 4), and the upregulation of mitochondrial ATP-synthesis related transcript (Fig. 6D), the comparison of day 50 hOCMTs also showed similar clusters on metabolic genes to adult heart, such as oxidative phosphorylation (*PPPARGC1A, MT-ATP6*)*,* fatty acid metabolism (*FABP3, GPAT3, PAM*), lipid metabolism (*FITM1, PRKAA2, INPP4B*)*,* and glycogen metabolism (*PPP1R3A*).

Finally, we compared the transcriptome of our 3D hOCMTs to Engineered Heart Tissue (EHT)^31,78^, a well-established model of 3D cardiac tissue obtained from hiPSCs. The analysis demonstrated that 3D long-term culture (day 50) prompted the expression of similar levels of genes encoding for sarcomere-associated proteins, which included *TTN*, *ACTN2*, troponins (*TNNT2*, *TNNI3K*, *TNNI3*), Myosin heavy chain *(MYH7*), calsequestrins (*CASQ2*) among many others (Supplementary Fig. 5).

Overall, transcriptomics analysis confirmed that our long-term 3D protocol induced hiPSCs cardiac specification and cardiomyocyte maturation at a metabolic, calcium handling and sarcomeric level. The maturation of 3D hOCMTs is strongly promoted by time in culture.

### Long-term human iPSC-derived organotypic cardiac microtissues display enhanced metabolic activity

The transcriptomic analysis of 3D hOCMTs was further exploited to investigate their metabolic maturation as compared to the 2D monolayers.

The heart is characterized by very high and distinct metabolic demands, with adult cardiomyocytes relying on oxidative phosphorylation for ATP production. On the contrary, immature hiPSC-derived cardiomyocytes display continued reliance on glycolysis as the primary source of energy^79^. Although the healthy heart can oxidize several substrates from energy production, classic studies estimate that ATP production comes preferentially from fatty acid (FA) oxidation, accounting for around 70% of the total generation. The rest is provided by alternative substrates, including glucose, lactate and pyruvate^80^.

**Figure 7:**
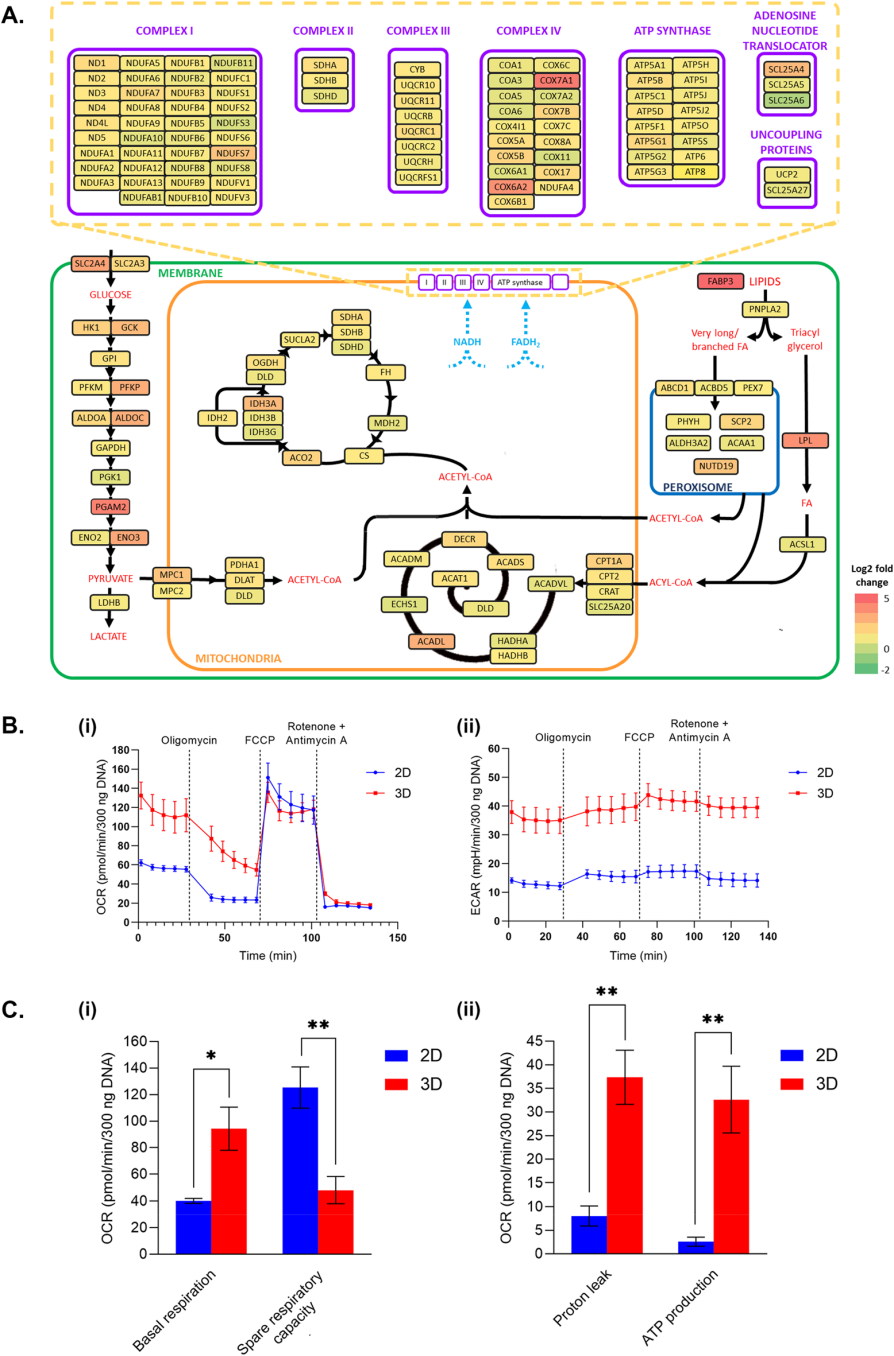
3D long-term cultures promote changes in metabolic activity towards maturation. (**A**) Diagram showing metabolic genes with significantly upregulated (in red) and downregulated (in green) genes between long-term 3D hOCMTs vs 2D monolayer cultures (log2fold change; p.adj < 0.05). (**B**) Graphs for comparing the cellular energy metabolism of long-term 3D hOCMTs vs 2D monolayer cultures after day 50 using Seahorse XFp Analyzer and Seahorse Mito Stress Test Kit: (i) oxidative phosphorylation (oxygen consumption rate, OCR) and (ii) glycolytic flux (extracellular acidification rate, ECAR) (Mean ± SEM obtained in four independent analyses. Total sample size: n_2D_ = 12, n_3D_ = 9). (**C**) Bar graphs providing a detailed analysis of the oxygen consumption rate (OCR) Seahorse XFp Analyzer data presented in (**B**): (i) basal respiration and spare respiratory capacity, (ii) proton leak, ATP production (Mean ± SEM, N = 4, n_2D_ = 12, n_3D_ = 9, unpaired Student’s *t* test—two-tailed).

Our 3D hOCMTs displayed higher expression of genes related to the FA β-oxidation, which has been previously associated with a more mature phenotype in hPSC-CMs (Fig. 7A)^81^. The expression of key regulator PPARA was—in fact—enhanced in the 3D culture. More directly into the lipid metabolism, the heart-type fatty acid binding protein (*FABP3*) and the lipoprotein lipase (*LPL*) displayed 4 times fold upregulation (Fig. 7A). The cytosolic fatty acyl-CoA synthase (*ACSL1*) catabolizing the long-chain acyl-CoA ester production, and the proteins required for their transfer into mitochondria (*CPT1A, CPT2, CRAT, SLC25A20*) were also found increased in the hOCMTs. Inside the mitochondria, most of the genes related to β-oxidation were found significantly upregulated as well, including the sequential acyl-CoA *dehydrogenases* (*ACDVL, ACDL, ACDM, ACDS*), which are highly expressed in the myocardium. The expression of several genes associated with peroxisomal FA intake and β-oxidation were also found upregulated.

Nevertheless, crucial genes related to glucose and pyruvate metabolism were also upregulated in the hOCMTs: this list included the glucose transporters *SLCA3* (GLUT3) and *SLCA4* (GLUT4), most of the enzymes associated with glycolysis, the proteins transporting the resulting pyruvate into the mitochondria (*MPC1, MPC2*), and its conversion into Acetyl-CoA (*PDHA1, DLAT, DLD*). Similarly, transcripts encoding for all the catabolic steps of the tricarboxylic acid cycle were more abundant in the 3D hOCMTs (Fig. 7A).

In accordance with the increased number of mitochondria (see Fig. 5A, Supplementary Fig. 3), this increase in mitochondrial metabolism was combined with an increase in the expression of genes associated to ATP synthesis-coupled electron transport. Most of the genes encoding for the proteins composing the four electron transport protein complexes were found upregulated, as well as the vast majority of the subunits of the adenosine triphosphate (ATP) synthase itself (Fig. 7A, up). Several of the mitochondrial genes associated with the electron transport chain were likewise enriched in the hOCMTs, including NADH-ubiquinone oxidoreductase chain subunits (ND1, ND2, ND3, ND4, ND4L, ND5) and ATP synthase subunits (ATP6, ATP8).

In order to confirm that 3D long-term (over 50 days) culture induces hiPSC metabolic maturation, we performed real-time analyses of 2D monolayer and 3D microtissue cultures’ energy metabolism using the Seahorse XFp Analyzer and Seahorse Mito Stress Test Kit. Results presented in Fig. 7B indicate a highly energetic phenotype of 3D hOCMTs compared to the 2D cultures. The 3D cultures not only exhibited a significantly higher oxygen consumption rate (OCR) than the 2D cultures (111.83 ± 17.21 vs 55.43 ± 2.82 pmol/min/300 ng DNA—the last baseline measurement before Oligomycin injection) but also increased extracellular acidification rate (ECAR, 35.09 ± 4.60 vs 12.19 ± 1.24 mpH/min/300 ng DNA—the last baseline measurement before Oligomycin injection).

A detailed analysis of the Mito Stress Test data revealed a significantly higher basal mitochondrial respiration in the 3D cultures (94.38 ± 16.28 vs 40.22 ± 1.89 pmol/min/300 ng DNA, p < 0.05; Fig. 7C). Interestingly, the 2D cultures showed a higher spare respiratory capacity than the 3D cultures (125.35 ± 15.52 vs 48.20 ± 10.18 pmol/min/300 ng DNA, p < 0.01; Fig. 7C). Notably, despite a relatively high dissipation of the mitochondrial proton gradient in hOCMTs (proton leak, 37.37 ± 5.75 vs 8.02 ± 2.13 pmol/min/300 ng DNA, p < 0.01; Fig. 7C), the 3D cultures produced significantly more ATP via mitochondrial respiration than their 2D counterparts (ATP production, 32.62 ± 7.08 vs 2.59 ± 0.95 pmol/min/300 ng DNA, p < 0.01; Fig. 7C).

Our transcriptomics and Seahorse XF metabolic flux data suggest that hOCMTs showed an enhanced metabolic activity, which was associated with increased metabolic signatures related to FA oxidation, mitochondrial respiration and glycolysis.

### Human iPSC-derived organotypic cardiac microtissues functionally respond to drugs in a dose dependent fashion in long-term culture

An essential feature of *bona fide* organoids is to replicate at least one specialized function of the modelled organ^51–53^, which in the case of heart tissue can be assessed by the contractile activity of human cardiac organoids (hCOs). As previously indicated, once our 3D hOCMTs acquired spontaneous contractile activity early in culture (48 h post aggregation), the contractility persisted for more than 100 days (Supplementary Video 2).

In order to test their physiological significance, 3D hOCMTs were exposed to clinically relevant doses of cardioactive drugs^82,83^ in long-term cultures (> day 50). In detail, isoproterenol and verapamil were used as positive and negative inotropes, respectively. Isoproterenol, a beta-adrenergic agonist, increases the contractile force and beating frequency^84^, while verapamil, a calcium channel blocker, decreases the beating rate^85^. The hOCMTs were incubated with increasing drug doses (0.01–1 µM) for 15–20 min and further monitored by live imaging using a confocal microscope.

**Figure 8:**
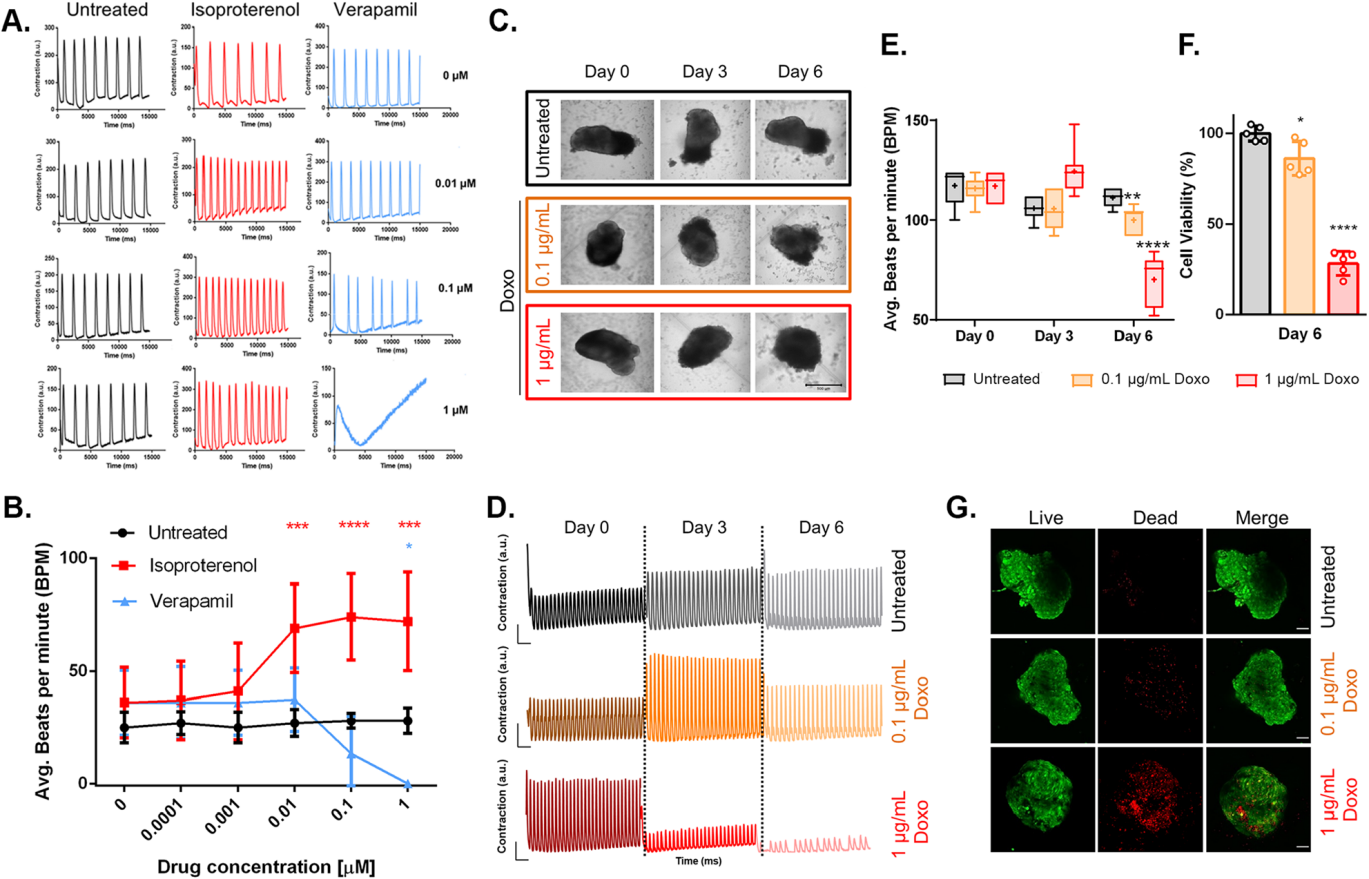
Long-term 3D hOCMTs show functional response to cardioactive and cardiotoxic drugs in a dose and time dependent manner. (**A**) Representative contraction amplitude plots of long-term (> day 50) Long-term 3D hOCMTs in response to untreated vs increasing doses of Isoproterenol and Verapamil. (**B**) Average beating rates of 3D long-term cardiac microtissues hOCMTs in response to increasing doses of Isoproterenol and Verapamil (n = 3, Mean ± SD, Dunnett’s multiple comparisons test). (**C**) Morphology of 3D long-term (> day 50) Long-term 3D hOCMTs in response to untreated vs increasing doses of Doxorubicin (Doxo) over 6 days (scale bar = 500 µm). (**D**) Representative contraction amplitude plots of Long-term 3D hOCMTs in response to untreated vs increasing doses of Doxo over 6 days (t = 15 s). (**E**) Average beating rates of Long-term 3D hOCMTs in response untreated vs increasing doses of Doxo over 6 days (n = 6, Lines = Median ± min/max, Dots = Mean value, Dunnett’s multiple comparisons test). (**F**) Luminescence-based quantification of cell viability Long-term 3D hOCMTs on day 6 of Doxo treatment normalized to untreated samples (untreated vs 0.1 µg/mL vs 1 µg/mL) (n = 6, Mean ± SD, Tukey’s multiple comparisons test). (**G**) Representative cell viability of v on day 6 of Doxo treatment (untreated vs 0.1 µg/mL vs 1 µg/mL) with Calcein/AM staining, green = live, red = dead (Scale bar = 100 µm).

The response to the treatment was evaluated and quantified based on the contraction (Fig. 8A) and beating rate (Fig. 8B) of the hOCMTs, analysed through the open-source software tool MUSCLEMOTION^86,87^. The acquired data shows that increasing concentrations of isoproterenol resulted in enhanced contraction amplitude and beating rate, with the beating rate peak at 1 µM. As expected, increasing concentrations of verapamil had the opposite effect, with the beating being completely hindered at 1 µM drug dosage (Supplementary Videos 3–8). These observations are in accordance with those reported in previous studies in cardiac microtissues^88^ and confirm that our constructs can functionally respond to cardioactive drugs in a dose dependent manner.

Finally, we investigated the possibility of modelling drug-induced cardiotoxicity on our hiPSC-derived microtissues, by treating them with doxorubicin (Doxo), a well-known chemotherapeutic agent widely used in the treatment of several types of cancers^89^. The drug is—in fact—also known to have cardiotoxic and pro-fibrotic effects, thus able to cause or exacerbate heart failure in vivo^90–95^.

The 3D hOCMTs were exposed to 0.1 µg/mL and 1 µg/mL of Doxo and their morphology and contractile activity was compared with untreated controls (n = 6) for the next 6 following days. The culture medium was exchanged every 3 days and the cultures were monitored on a daily basis. Figure 8C shows that the morphology of the constructs gradually changed over the course of treatment, and with the occurrence of more irregular edges when exposed to higher Doxo doses. In addition, clear changes could also be observed in the beating profiles of the constructs treated with Doxo (Fig. 8D,E, and Supplementary Videos 9–14).

We figured the changes in the morphology of the microtissues treated with the chemotherapeutics might be due to the induction of cell death by the drug. Therefore, after 6 days of Doxo treatment, we assessed the viability of the constructs with a luminescence-based assay. The results in Fig. 8F,G revealed that the 3D hOCMTs treated with 1 µg/mL Doxo were significantly less viable than both the control group and the group treated with lower drug concentration (0.1 µg/mL) (Average viability on day 6: 100 ± 4.1% for ctrl, 86.4 ± 9.3 for 0.1 µg/mL Doxo, 28.3 ± 6.6% for 1 µg/mL Doxo, Mean ± SD, p < 0.0001).

## Discussion

In this study we present a simple method to generate 3D iPSC-derived human cardiac microtissues in the absence of an external scaffold that could be cultured for extended periods.

Our study has demonstrated that long-term cultured 3D cardiac microtissues reveal some characteristics resembling native human heart, such as: (1) spontaneous 3D organization in scaffold-free conditions; (2) presence of multiple cell types of the heart with a tendency to re-organize into distinct and discrete structures; (3) ultrastructural organization and maturation of cardiac muscle sarcomeres and mitochondria; (4) improved survival, cardiac specificity and maturation at transcriptional and metabolic levels; (5) long-term functional beating in the absence of external stimuli; (6) functional response to cardioactive and cardiotoxic drugs in a dose- and time-dependent fashion. Given that long-term cultured 3D cardiac microtissues possess the abovementioned characteristics, we propose that the hOCMTs can be defined as human cardiac organoids (hCOs).

When compared to standard 2D cardiomyocytes culture, 3D hOCMTs showed an enhanced survival rate. More importantly, their cellular composition appeared to be more representative of the physiological conditions in the heart, although their overall phenotype appeared to be close to foetal heart^88,96^.

While the effects of long-term culture in promoting the maturation of PSC-derived cardiomyocytes is well established^69,97^, 2D culture is usually challenging in the long run, as the cardiomyocytes tend to delaminate from the traditional tissue culture plates^69^, leaving non-myocyte cell populations such as fibroblasts to overgrow.

One of the greatest advantages of our model is that there is no need for an external scaffold as the microtissues self-organize and survive for a long time in static culture conditions. Other models have been developed in the absence of an external ECM, however they usually require the differentiation of other stromal cells (such as epicardial or fibroblastic cells) separately, followed by their assembly with cardiac myocytes^35,56^.

On the contrary, by exploiting the limited efficiency of the original 2D cardiac differentiation protocol by Lian et al.^13,14^ and simply switching the dimensionality from 2 to 3D, we have generated long-term 3D hOCMTs in which epicardial cells, fibroblasts, and endocardial cells emerge spontaneously together with cardiomyocytes, without the need to set up separate lineage differentiation protocols and further assembly. In addition, the spontaneous beating of our hOCMTs was observed for more than 100 days as, to this date, only reported by Silva et al. as well. The latter paper, though, reported that the developmental co-emergence of gut also occurred together with the heart tissue^65^.

In our model, a distinct gut tissue was not observed after 50 days of culture through immunofluorescence analysis. Histological analysis of the hOCMTs has shown distinct populations of WT1-, TBX18-positive epicardial cells, TE-7-, SM22α-positive fibroblast-like cells, NFAT2-positive endocardial cells along with a distinct core of cardiomyocytes. CD31-positive endothelial cells could also be detected. The low number of CD31- positive cells might be explained by the fact that in native human heart endothelial cells have different developmental origins^98^, and we did not supplement our culture media with endothelial-lineage favouring factors, like VEGF, during our study.

Another interesting feature of the hOCMTs is the occurrence of cardiomyocytes expressing both atrial and ventricular markers, as identified by confocal imaging of MYL2 and MYL7. Although MYL7 is known to be observed on all cardiomyocyte subtypes during development^74^, we could identify cardiomyocytes that are MYL7 positive, but not MYL2 positive, or vice versa, suggesting there could be a tendency of commitment to formation of different cardiomyocyte subtypes. Despite significant differences, a similar phenomenon of atrial/ventricular cardiomyocyte subtype distinction was also shown by Israeli and colleagues, which appeared to be blunted with long-term culture^64^. Instead, the herein reported hOCMTs revealed higher cardiac-specific cellular heterogeneity and maturation in terms of histological, ultrastructural, and transcriptional-levels in long-term 3D cultures compared to shorter culture times and standard 2D monolayer culture.

The maturity and complexity of our construct could be shown at the ultrastructural level by our TEM analysis over long culture times. On day 50 and 85, our hOCMTs displayed clearly aligned myofibers resulting in well-organized sarcomeres, as compared to earlier time points. These morphological adaptations, which are hallmarks of mature cardiac tissues^67^, were further supported by transcriptomics analysis, where hOCMTs showed an increased expression of genes related to cardiac contraction and sarcomeric structures. The maturity of our long-term 3D hOCMTs could also be demonstrated by comparison with Engineered Heart Tissues (EHTs), a well-recognized 3D model of cardiac tissue in vitro, by showing our 3D constructs share with EHTs a similar transcriptional landscape.

The calcium handling properties of immature cardiomyocytes diverge from their mature counterpart. While immature cardiomyocytes can rely on the calcium released at the cell periphery to initiate sarcomere contraction, mature cardiomyocytes develop plasma membrane invaginations (t-tubules) which are wrapped around the myofibrils as to make the process of calcium release and re-uptake faster and more efficient^99,100^. The first polarization triggers the release of calcium stored in the sarcoplasmic reticulum by RYR2, which is pumped back to the sarcoplasmic reticulum by SERCA2 (sarco/endoplasmic reticulum Ca^2+^-ATPase). The analysis of hOCMTs confirmed the presence of t-tubules at the longer time-points (> day 50), the upregulation of some of the genes involved in their biogenesis (*JPH2*, *ACTN2*, *NEXN*) and sarcomeric calcium management (*RYR2*, *PLN*, *SERCA*), hence demonstrating long-term 3D culture determines functional maturation of hOCMTs.

Furthermore, cardiomyocytes also experience several metabolic adaptations during in vivo maturation in order to generate the required amount of ATP^74,101^. Our ultrastructural results are compatible with this metabolic switch, showing that hOCMTs acquired elongated mitochondria distributed around the contractile apparatus with time in culture. This idea is further reinforced by the upregulation of genes related to electron transport chain-linked phosphorylation and FA β-oxidation in long-term 3D cultured hOCMTs, which has been previously linked to a more mature phenotype in hPSC-CMs^81^. Interestingly, the increase in electron transport chain activity has been linked to the cardiomyocyte differentiation and maturation not only through metabolic support, but also by generating calcium oscillations though complex III activity^102^. Recent protocols have been devised that improve pluripotent stem cell-derived cardiomyocyte metabolic maturation, and push them closer to adult cardiomyocytes, which rely on oxidative phosphorylation for ATP production^79^.

Our analyses of cell energy metabolism using the Seahorse XF technology revealed hOCMTs as highly energetic compared to the 2D cultures. The 3D hOCMTs consumed much more oxygen, a significant proportion of which was used for mitochondrial ATP production. The proton leak in the mitochondria of hOCMTs might serve as a protective mechanism against mitochondrial damage by limiting reactive oxygen species production^103^. The surprisingly similar levels of maximal respiration in the 3D and 2D cultures induced by FCCP stimulation indicated that the mitochondria of 2D culture cells were also metabolically competent and possessed a high respiratory capacity. However, their respiratory capacity was only partially used for mitochondrial ATP production under standard culture conditions. We also observed an increased extracellular acidification rate in the 3D cultures that nicely correlated with the results of transcriptomics analyses showing a general upregulation of metabolic gene expression, including glycolysis genes. This increase in glycolysis might reflect individual metabolic adaptations of various cell types and subpopulations in hOCMTs and could potentially be linked to differential oxygen availability inside hOCMTs. Together, our Seahorse XF analyses showed that cellular energy metabolism strongly differs in the 2D and 3D cultures, and the 3D hOCMTs relied significantly more on mitochondrial respiration to satisfy their energy needs.

Functional analysis of the 3D hOCMTs proved that they were responsive to clinically relevant doses of cardioactive and cardiotoxic drugs. This is the first step required to consider the generation of patient-specific models of disease for drug testing.

As for modelling chemotherapy-induced cardiotoxicity, while doxorubicin is a well-established chemotherapeutic known to cause heart failure as a side effect^90–95^, the in vitro modelling in cardiac microtissues is still not well-established due to the limitations so far encountered in reproducing the physiological cellular heterogeneity, and cardiomyocyte maturity^62^. Furthermore, it is crucial to consider the influence of cell–cell interactions of cardiomyocyte vs non-myocytes—especially fibroblasts—and cell-ECM interactions^104^. Our hOCMT platform offers the advantages of long-term viability, cellular heterogeneity and advanced maturity in vitro, rendering it a physiologically relevant alternative for future studies. For instance, further clinical parameters such as the cardio-fibrotic potential of doxorubicin treatment^93,94,105^, the secretion of cardiac troponins and natriuretic peptides as clinically relevant markers of chemotherapy-induced cardiotoxicity^106^ or simply the potential for personalized medicine could be exploited by using this iPSC-derived hOCMT^107^.

Although promising, our study shows some limitations. Despite the structural and functional maturation in the long-term culture model as compared to traditional monolayers, hOCMTs still display features of the foetal rather than the adult heart. For example, myocytes of our hOCMTs have ~ 1.5 µm length of sarcomeres (as in foetal mammalian hearts), whereas in adult hearts, this length is usually 2 µm^67^. We believe the adoption of additional stimuli (i.e.: dynamic culture, mechanical conditioning and electrical stimulation) might in the future help further improve the model here described^108^. Furthermore, despite showing hints of histological similarities to the heart tissue, cellular heterogeneity of cardiac cell types, and spontaneous re-organization of these multiple cell types at distinct regions, additional cues would be needed to establish true self-organization following the same principles of embryonic human heart development.

In a longer perspective, in vitro cardiotoxicity studies should not underestimate the importance of multi-organ interactions. While drugs in their native chemistries may not always result in direct cardiac damage^109^, their metabolic by-products might cause cardiotoxicity in vivo^109,110^.

In conclusion, we have established a simple procedure to prepare human induced pluripotent stem cell-derived organotypic cardiac microtissues. We propose that hOCMTs could be considered as cardiac organoids, for their ability to recapitulate different key features of the human heart, and display improved maturation and functionality in long-term culture, thus enhancing the potential of in vitro platforms to be used in translational research applications.

## Methods

### Stem cell culture and maintenance

The human iPSC cell line DF 19-9-7 T (iPS, karyotype: 46, XY) was purchased from WiCell (Madison, WI, USA), and the Troponin I1 reporter iPSC line (TNNI1-iPS) was purchased from Coriell Institute (Cat. no. AICS-0037-172, Camden, New Jersey, USA). All iPSC cells were cultured and maintained in feeder-free conditions as previously described^111,112^ on Growth Factor Reduced Matrigel^®^-coated plates, (1:100 in DMEM/F12, Corning) in complete Essential 8™ Medium (E8, Thermo Fisher Scientific) containing penicillin/streptomycin (P/S) (0.5%, VWR), incubated at 37 °C, 5% CO_2_.

### hiPSC-derived monolayer cardiac differentiation and maintenance

Cardiac differentiation from hiPSCs was performed following the Wnt signalling modulation protocol by Lian et al.^13,14^, with slight modifications as previously described^111^. Briefly, prior to cardiovascular differentiation, hiPSCs were dissociated into single cells (TrypLE Select, Thermo Fisher Scientific) and re-seeded onto 12-well Matrigel-coated plates with 2.0 × 10^5^ cells/cm^2^, with complete Essential 8 medium including Y27632 Rock Inhibitor (RI) (1:4000 dilution from 10 μM stock, 2.5 μM final, Selleck chemicals, Houston, TX, USA). The next day, the medium was replaced with complete Essential 8 medium without RI, and the medium exchange was performed daily until the cells reached 100% confluency. On day 0, to start mesoderm differentiation, the medium was changed with RPMI 1640 with L-Glutamine (Biosera) media supplemented with P/S, B-27™ supplement minus insulin (1 ×, Thermo Fisher Scientific) and CHIR99021 (8 µM, Sigma-Aldrich). On day 2, the medium was exchanged with RPMI 1640 + B-27 minus insulin (RPMI + B27-Ins), supplemented with IWP-2 (5 µM, Selleck chemicals). On day 4, the medium was replaced with RPMI + B27-Ins, and medium exchange was performed every 2 days until the cells started beating (usually on day 7, but may depend on the iPSC line). Once beating clusters started to emerge, the medium was replaced with RPMI 1640 + B-27™ supplement (RPMI + B27 + Ins) (1 ×, Thermo Fisher Scientific), and medium exchange was performed every 2–3 days until day 15 of differentiation. From day 15 on, the cells were used either for generation of 3D hOCMTs or were continued to be cultured as 2D monolayers as controls, with medium exchange every 3–4 days until the end of experiments.

### hiPSC-derived 3D organotypic cardiac microtissue (hOCMT) generation and maintenance

On day 15 of monolayer cardiac differentiation, cells were dissociated by putting 0.5 mL StemPro™ Accutase™ (Gibco) per well, and incubating in room temperature or in 37 °C incubator for 10–20 min with occasional mechanical pipetting, until the cells were visually dissociated. After sufficient dissociation, Accutase™ was stopped by putting 3 mL RPMI 1640 with 20% Knockout Serum Replacement (Gibco) per well, and the cells were pelleted by centrifugation at 150×*g* for 3 min. The resulting pellet was resuspended with RPMI/B27 + Ins + P/S + RI (1:2000 dilution from 10 μM stock, 5 μM final), counted by LUNA™ cell counter (Logos), and seeded 150,000 cells/well in 96-well round bottom ultra-low attachment plates (Corning Costar 7007) at a volume of 150 µL/well. The plates were then centrifuged at 200×g for 5 min and incubated at 37 °C, 5% CO_2_. As 2D control, either 200,000 cells per well were seeded in Matrigel-coated 96-well tissue culture plates at a volume of 200 µL/well, or 1.5 × 10^6^ cells were seeded on Matrigel-coated 24-well tissue culture plates in 1 mL volume per well, or left undetached in 12-well plates. After 48 h, medium was replaced with RPMI/B27 + Ins without RI from hOCMTs and 2D-controls that were re-seeded. From this point on, medium was changed partially every 2–3 days with RPMI/B27 + Ins + P/S until day 30, then every 3–4 days at least until day 50, or longer.

### Cell viability with Calcein AM/EthD-1 staining

3D hOCMT viability was assessed with LIVE/DEAD™ Viability/Cytotoxicity Kit, for mammalian cells (Thermo Fisher Scientific), according to manufacturer’s instructions, and confocal imaging was performed with Zeiss LSM 780 confocal microscope.

### Terminal deoxynucleotidyl transferase (TdT) dUTP nick-end labeling (TUNEL) assay

The presence of 3D hOCMT apoptotic/necrotic core was assessed on hOCMT cryosections with Click-iT Plus TUNEL Assay Kit with Alexa Fluor 647 fluorophore (Thermo Fisher Scientific) according to manufacturer’s instructions. Positive control of cell death was performed by DNAse I treatment according to manufacturer’s instructions (Thermo Fisher Scientific, Cat. no. 18068015). Confocal imaging was performed with Zeiss LSM 780 confocal microscope.

### Immunofluorescence (IF)

3D hOCMT fixation, cryosectioning, and immunostaining was modified from Perestrelo et al.^104^. Briefly, 3D hOCMT s were collected with low binding pipette tips, and fixed in 4% PFA (Santa Cruz Biotechnology) supplemented with 0.03% eosin (Sigma Aldrich) for 2 h at room temperature, followed by washing with 1×PBS (Lonza), and embedding in 30% sucrose solution at 4 °C until they sink at the bottom (~ 2 days). The hOCMTs were then embedded in OCT solution (Leica), frozen in cassettes embedded in isopentane (VWR) cooled with dry ice, and were stored in − 80 °C until further processing. The frozen hOCMTs were cryosectioned by cryotome (Leica CM1950) onto Menzel Gläser, SuperFrost^®^ Plus slides (Thermo Fisher Scientific) at 10 µM thickness.

For IF analysis, the hOCMT sections were washed with PBS for 2 × 5 min at RT, followed by permeabilization with 0.2% Triton X-100 (Sigma Aldrich) for 5 min. Blocking was done by 2.5% bovine serum albumin (BSA) (Biowest) in PBS for our at room temperature. Primary antibodies were incubated overnight at 4 °C (SI—Supplementary Table 1). The next day, the samples were washed with PBS, followed by appropriate Alexa-conjugated secondary antibodies (SI—Supplementary Table 1). Counterstaining was done by DAPI (Roche), and hOCMT slides were mounted with Mowiol^®^4-88 (Sigma Aldrich). Confocal imaging was performed with Zeiss LSM 780 confocal microscope.

Further histological procedures can be found in Supplementary Methods.

### Microscopy and image analysis

Confocal imaging was performed with Zeiss LSM 780 confocal microscope. Light microscopy imaging was performed with Leica DM IL LED inverted microscope, and fluorescence images were collected with Leica DMI4000 B inverted fluorescence microscope. Image analysis was performed with ImageJ (v1.53o).

### 3D hOCMT dissociation

For flow cytometry and RNA isolation, hOCMTs were collected in Eppendorf tubes with low binding pipettes on day 30 and 50, and dissociated with MACS Multi Tissue Dissociation Kit 3 (Miltenyi Biotec, Cat. no. 130-110-204) according to manufacturer’s instructions. As controls, 2D monolayer cultures were directly dissociated on the plates with the same kit.

### Flow cytometry

Flow cytometry analyses on 3D hOCMTs and 2D controls were performed on day 15 (for 2D), day 30 (for 2D and 3D) and day 50 (for 2D and 3D) timepoints as described previously^104,112^ with modifications. Briefly, on each timepoint, samples were dissociated with MACS Multi Tissue Dissociation Kit 3, washed with FACS buffer (99.4% PBS + 0.1% UltraPure™ 0.5 M EDTA pH 8.0 by Invitrogen™ + 0.5% FBS by Sigma Aldrich [Cat. no. F9665-500ML]) and surface marker staining with CD90-APC (Exbio; 1:10 dilution in FACS buffer) and CD31-BV421 (Biolegend; 1:10 dilution in FACS buffer) antibodies was performed together with corresponding unstained controls for 30 min on ice. Afterwards, sample fixation and permeabilization was performed with Intracellular Fixation and Permeabilization Buffer Set (eBiosciences) per manufacturer’s instructions, followed by intracellular marker staining with TNNT2-FITC (Miltenyi Biotec; 1:50 dilution in 1 × Permeabilization Buffer) antibody together with corresponding unstained controls for 30 min in room temperature. Sample acquisition was performed by using BD FACSCanto II (Becton, Dickinson and Company, Franklin Lakes, NJ, USA), and cell populations were analysed with FlowJo v.10 (Tree Star) (see SI—Supplementary Table 1 for detailed antibody information).

### Transmission electron microscopy (TEM) sample preparation and analysis

3D hOCMTs were transferred to new well-plates with low binding pipettes, and washed 2 × with PBS. Fixation was done by 1.5% PFA + 1.5% glutaraldehyde (Sigma Aldrich) diluted in RPMI 1640 culture media for 1 h, then washed with 0.1 M cacodylate buffer (pH 7.4) (Sigma Aldrich) for 3 times 5 min each, and incubated overnight at 4 °C in cacodylate with glutaraldehyde. The next day, samples were rewashed 3 times with cacodylate buffer, and incubated at 4 °C in cacodylate buffer until sending the samples, protected from light. The 3D hOCMTs were post-fixed for 1.5 h with 1% osmium tetroxide in 0.1 M cacodylate buffer and washed for 3 times with 0.1 M cacodylate buffer, 10 min each, then stained with 1% uranyl acetate in milli-Q water overnight at 4 °C, followed by washing in milli-Q water. The samples were then dehydrated in an ascending EtOH series using solutions of 70%, 90%, 96% and 3 times 100% for 10 min each, incubated in propylene oxide (PO) 3 times for 20 min before incubation in a mixture of PO and Epon resin overnight, and incubated in pure Epon for 2 h and embedded by polymerizing Epon at 68 °C for 48 h. Ultra-thin sections of 70 nm were cut using a Leica Ultracut EM UC 6 Cryo-ultramicrotome. TEM images were collected with a JEOL JEM 1011 electron microscope and recorded with a 2 Mp charge coupled device camera (Gatan Orius).

### RNA isolation, bulk RNA-sequencing and DE analysis

For RNA isolation, 3D hOCMTs and 2D controls were dissociated by using MACS Multi Tissue Dissociation Kit 3 on day 30 and day 50. After cell dissociation, RNA was extracted with High Pure RNA Isolation Kit (Roche) according to manufacturer’s instructions, and quantified with Nanodrop 2000 Spectrophotometer (Thermo Fisher Scientific). Quality control for RNA integrity number (RIN) was also determined with Agilent 2100 Bioanalyzer.

Bulk RNA-sequencing and DE analysis procedures are further described in Supplementary Methods.

Data were deposited in the NCBI Gene Expression Omnibus repository with the GEO ID GSE209997.

Gene ontology (GO) enrichment analysis for biological processes was performed with EnrichR web-tool^113–115^. Clustering analysis of upregulated genes was done with Cytoscape^116^.

RNA-seq data from this study were also compared to adult human heart RNA-seq data (from BioProjects PRJNA667310^76^ and PRJNA628736^77^), and EHT RNA-seq data (from BioProject PRJNA831794^78^) obtained from the NCBI BioProject database (https://www.ncbi.nlm.nih.gov/bioproject/) (Accession numbers: SRR12771050, SRR12771052, SRR11620685, SRR11620686, SRR18911472, SRR18911473, SRR18911476, SRR18911477) by using Biojupies web-tool^117^.

### Metabolic flux analysis

Mitochondrial respiration of 3D hOCMTs and 2D controls was assessed using Seahorse XFp Analyzer (Agilent) with Seahorse XFp Cell Mito Stress Test Kit (Agilent). On the Day 50 timepoint, 8-well XFp Cell Culture Miniplates (Agilent) were coated with Matrigel (1:20 dilution), and both 3D hOCMTs (one centered 3D hOCMT per well) and 2D controls (dissociated with MACS Multi-tissue dissociation kit 3 and seeded at 200,000 cells/well) were plated. The miniplates were cultured for 48 h in RPMI/B27 + Ins + P/S + RI (1:2000 dilution from 10 μM stock, 5 μM final) media to allow stabilization, and for the next 24 h in RPMI/B27 + Ins + P/S media. For 3D hOCMTs, only beating samples located at the center of the well were included in the subsequent statistical analysis.

One hour before measurement, RPMI/B27 + Ins + P/S media was replaced with Seahorse XF RPMI Medium pH 7.4 (Agilent) supplemented with 10 mM glucose, 2 mM glutamine, and 1 mM pyruvate (Agilent). Metabolic inhibitors were diluted in the supplemented XF Medium, loaded into the injection ports of the hydrated Seahorse XFp Sensor Cartridge (Agilent), and injected at specified time points to the wells as follows: Oligomycin (3 µM), FCCP (4 µM), and Rotenone/Antimycin A (2 µM). The Mito Stress Test was performed according to the manufacturer's instructions and included five measurements for basal consumption and five measurements after every drug injection. An extra five-minute waiting step was included after the Oligomycin injection.

After the measurement, both 3D hOCMTs and 2D controls were dissociated with MACS MultiTissue Dissociation Kit 3 (Miltenyi Biotec), and lysed for 2 h at 60 °C (10 mM Tris, 1 mM EDTA, 50 mM KCl, 2 mM MgCl_2_, 200 µg/mL Proteinase K as described previously by Giacomelli et al.^35^. Metabolic data were normalized by measuring the DNA content of each sample with a Quant-iT™ PicoGreen™ dsDNA Assay Kit (Thermo Fisher Scientific) according to the manufacturer's protocol. Wave software (Agilent), Microsoft Excel, and Prism 9 (GraphPad Software) were used for data analysis.

### Drug treatments

Isoproterenol hydrochloride (Sigma Aldrich) was dissolved in dimethyl sulfoxide (DMSO) (Sigma Aldrich), and Verapamil hydrochloride (Sigma Aldrich) in methanol (VWR) to prepare 1 M stock solutions. Each drug solution was 10- fold serially diluted in RPMI/B27 + Ins media to make final concentrations between 0.0001–1 µM. As untreated controls, complete RPMI/B27 + Ins media supplemented with DMSO or methanol were used. For data collection, the 3D hOCMTs with drug-supplemented media were incubated for 10 min at 37 °C, 5% CO_2_ each time the drug concentration changed, followed by live imaging with video acquisition per condition with Zeiss LSM 780 confocal microscope. Chemotherapy-induced cardiotoxicity was evaluated in 3D hOCMTs treated with RPMI/B27 + Ins supplemented with doxorubicin (Doxo) (Sigma Aldrich), at 0.1 µg/mL and 1 µg/mL concentrations, or plain RPMI/B27 + Ins as control for 6 days. Growth medium was exchanged every 3 days, and 3D hOCMT morphology and beating were monitored daily by live video recording with Zeiss LSM 780 confocal microscope for 6 days. On day 6 of drug treatment, the 3D hOCMTs were collected for evaluating viability.

### Live video acquisition and contraction analysis

To evaluate the contractile function of 3D hOCMTs, live image sequences were acquired with Zeiss LSM 780 Confocal microscope using transmitted light mode (37 °C, 5% CO_2_), as equivalent to 60 fps for 15 s; where the image sequences were later turned into .avi files with ImageJ. The beating rates were counted and calculated manually from the video analysis, and representative contraction graphs were derived with the open-source video analysis software MUSCLEMOTION^86,87^ per providers’ instructions.

### Luminescence-based cell viability assay

3D hOCMTs viability after 6 days of Doxo treatment was assessed by CellTiter-Glo^®^ Luminescent Cell Viability Assay Kit (Promega) according to manufacturer’s instructions. After the 3D hOCMTs were dissociated by the assay treatment, the samples were transferred to a clear-bottom 96-well white assay plate (Corning Costar 3610) and cell viability was quantified with Centro LB 960 Microplate Luminometer (Berthold Technologies).

### Statistics

Data are expressed as mean ± SD or mean ± SEM where applicable, unless otherwise stated. Statistical analyses were performed using Microsoft Excel 2010 and GraphPad Prism 8.0.2 unless otherwise stated, using one-way ANOVA (with Tukey’s multiple comparisons test), two-way ANOVA (with Dunnett’s multiple comparisons test), or unpaired Student’s *t* test (two-tailed) where p < 0.05 was considered to be significantly different as denoted with asterisks [*p ≤ 0.05, **p ≤ 0.01, ***p ≤ 0.001, ****p ≤ 0.0001]. Sample sizes of independent experiments, statistical methods used and the significance were described in the figure legends wherever applicable.

## Supplementary Information

Supplementary Information 1.

Supplementary Information 2.

Supplementary Information 3.

Supplementary Video 1.

Supplementary Video 2.

Supplementary Video 3.

Supplementary Video 4.

Supplementary Video 5.

Supplementary Video 6.

Supplementary Video 7.

Supplementary Video 8.

Supplementary Video 9.

Supplementary Video 10.

Supplementary Video 11.

Supplementary Video 12.

Supplementary Video 13.

Supplementary Video 14.

## Data Availability

The RNA-sequencing data obtained and presented in this study were generated at CF Genomics of CEITEC, and is available in the NCBI GEO public repository with accession no. GSE209997 (https://www.ncbi.nlm.nih.gov/geo/query/acc.cgi?acc=GSE209997). The data that support the findings of this study are available from the corresponding author upon reasonable request.
