# Supplementary material for: Generation and maturation of human iPSC-derived 3D organotypic cardiac microtissues in long-term culture
Source: Sci Rep. 2022 Oct 18;12:17409. doi: 10.1038/s41598-022-22225-w (PMC9579206; doi:10.1038/s41598-022-22225-w)
Supplement: Supplementary file 1 — Supplementary Information 1. [file 41598_2022_22225_MOESM1_ESM.pdf]

SUPPLEMENTARY INFORMATION

Generation and Maturation of Human iPSC-derived 3D Organotypic Cardiac Microtissues in Long-Term Culture

Ece Ergir[1,2], Jorge Oliver-De La Cruz[1], Soraia Fernandes[1], Marco Cassani[1], Francesco Niro[1,3], Daniel Pereira-Sousa[1,3], Jan Vrbský[1], Vladimír Vinarský[1], Ana Rubina Perestrelo[1], Doriana Debellis[4], Natália Vadovičová[3], Stjepan Uldrijan[3], Francesca Cavalieri[5,6], Stefania Pagliari[1], Heinz Redl[7,8], Peter Ertl[2,8], and Giancarlo Forte[1,9]

[1] Center for Translational Medicine (CTM), St. Anne's University Hospital, International Clinical Research Centre (FNUSA-ICRC), CZ-62500, Brno, Czech Republic
[2] Faculty of Technical Chemistry, Institute of Applied Synthetic Chemistry & Institute of Chemical Technologies and Analytics, Vienna University of Technology, AT-1040, Vienna, Austria
[3] Faculty of Medicine, Department of Biomedical Sciences, Masaryk University, CZ-62500, Brno, Czech Republic.
[4] Electron Microscopy Facility, Fondazione Istituto Italiano Di Tecnologia, Via Morego 30, IT-16163, Genova, Italy
[5] Department of Chemical Engineering, The University of Melbourne, Parkville, Victoria 3010, Australia;
[6] Dipartimento di Scienze e Tecnologie Chimiche, Università degli Studi di Roma Tor Vergata, via della Ricerca Scientifica 1, 00133, Rome, Italy
[7] Ludwig Boltzmann Institute for Experimental and Clinical Traumatology, AUVA Research Center, AT-1200, Vienna, Austria
[8] Austrian Cluster for Tissue Regeneration, AT-1200, Vienna, Austria
[9] Department of Biomaterials Science, Institute of Dentistry, University of Turku, FI-20014, Turku, Finland

Corresponding author:

Giancarlo Forte, PhD
Center for Translational Medicine (CTM)
International Clinical Research Center (ICRC)
St. Anne's University Hospital
Studentska 6, Brno
Czech Republic, 62500
Tel: +420-543185449
giancarlo.forte@fnusa.cz

Supplementary Figures

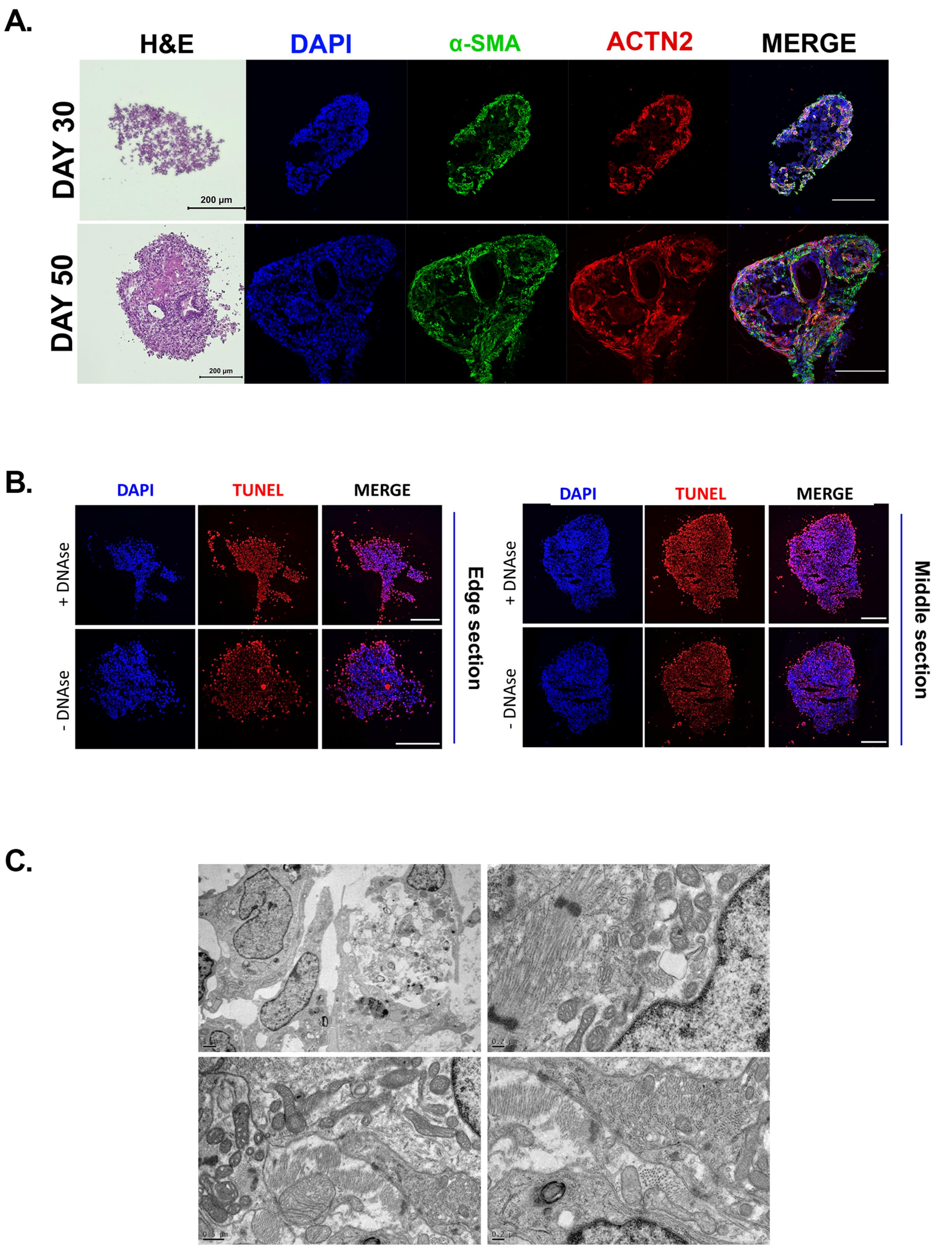

Figure SI-1 - 3D hOCMTs recapitulate advanced morphology and cellular heterogeneity in longer culture times

A. H&E staining and immunofluorescence analysis showing the increasing histological complexity of 3D hOCMT sections on day 30 vs day 50. Markers for cardiomyocytes (ACTN2 - red), smooth muscle/fibroblastic cells (α-SMA - green), shown, counterstained with DAPI (blue). Scale bars = 200µm
B. TUNEL staining (red) on day 30 3D hOCMT cryosections, with DAPI counterstain (blue) (edge and center sections) Scale bar = 200 µm
C. TEM images of day 50 hOCMTs ultrastructural organization, depicting viable and necrotic areas. Scale bars: top left = 1 µm, top right = 0.2 µm, bottom left = 0.5 µm, bottom right = 0.2 µm.

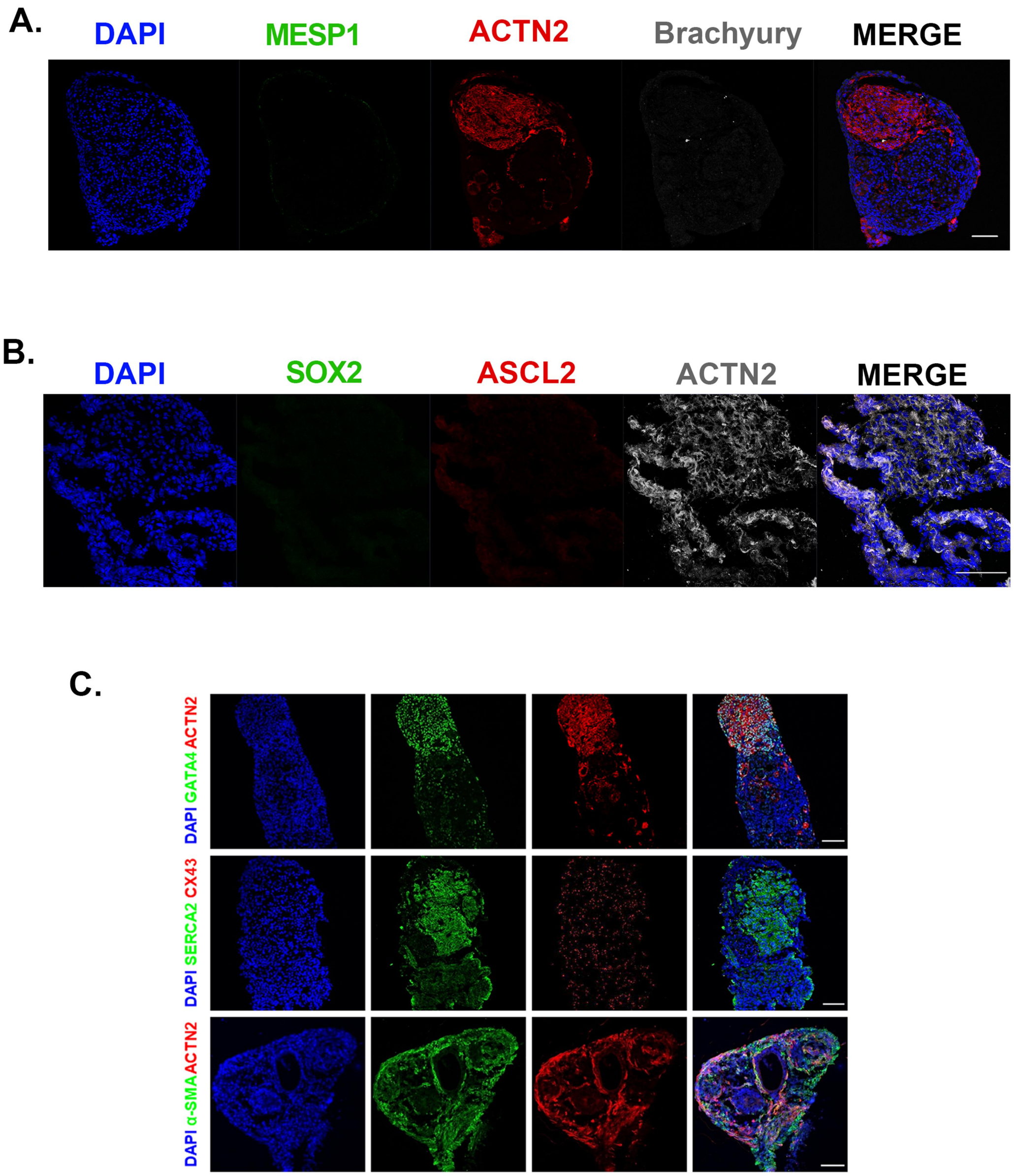

Figure SI-2 – Long-term hOCMTs show cardiovascular cellular heterogeneity and do not show markers for undifferentiated cells or co-emergence of gut tissue

A. IF staining of hOCMT sections for undifferentiated cell markers including MESP1 (green) and Brachyury (grey), compared to differentiated cardiomyocytes (ACTN2 - red) on day 50. Counterstaining with DAPI (blue). Scale bar = 100µm

B. IF staining of hOCMT sections for undifferentiated cell & embryonic gut (SOX2 - green), and adult gut (ASCL2 - red) tissue markers, compared to differentiated cardiomyocytes (ACTN2 - grey) on day 50. Counterstaining with DAPI (blue). Scale bar = 100µm

C. IF staining of hOCMT sections for cardiomyocytes (ACTN2 - red), cardiac morphogenesis (GATA4 -green), calcium ATPase (SERCA2 - green), gap junction proteins (CX43- red), and smooth muscle/fibroblastic cells (α-SMA - green). Counterstaining with DAPI (blue). Scale bar = 100µm

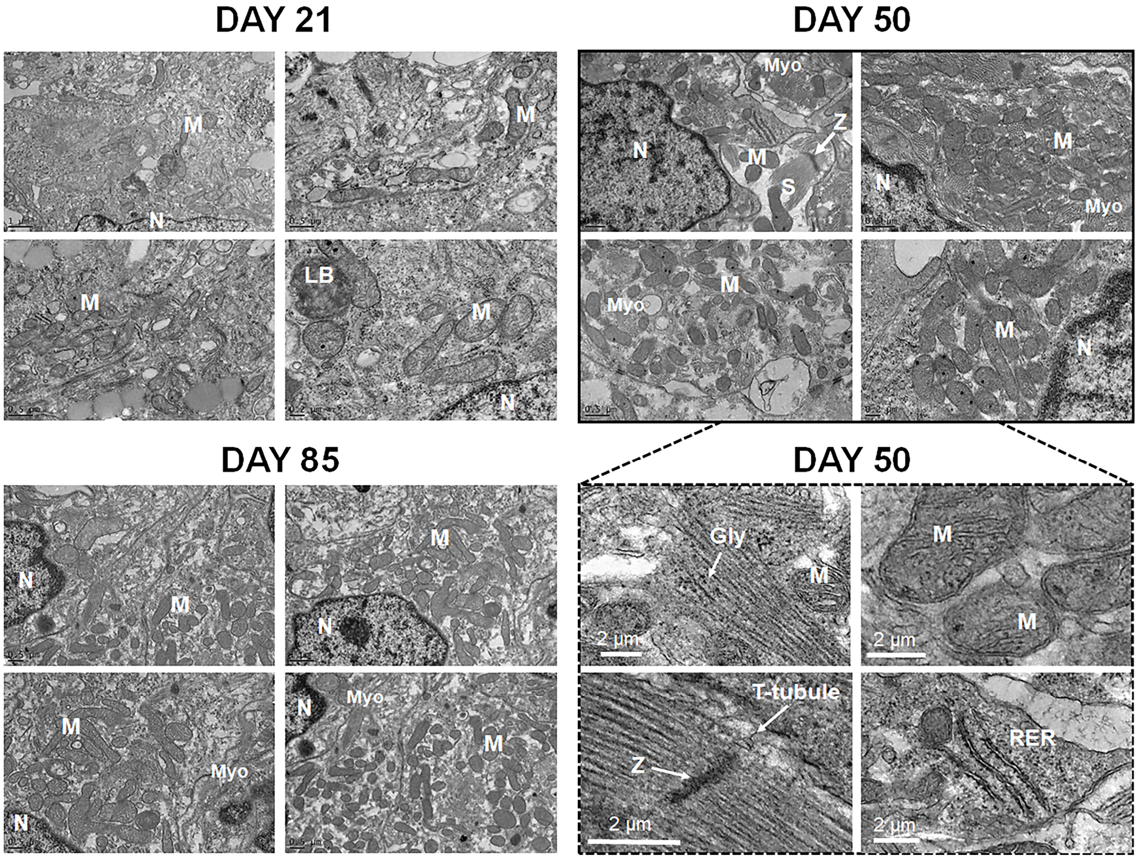

Figure SI-3 – Ultrastructural analysis of long-term hOCMTs indicates enhanced cardiomyocyte and metabolic maturation in 3D environment over time

TEM images of hOCMTs increasing ultrastructural organization and structural & metabolic maturity over time, from day 21 to day 85: Depicted: Cardiomyocyte myofibers (Myo), Sarcomeres (S), Mitochondria (M) Z-band (Z), T-tubules, Nuclei (N), Rough endoplasmic reticulum (RER), Glycogen granules (Gly), Lamellar bodies (LB). Scale bars are 1 µm and 0.5 µm for day 21, 0.5 µm for day 85, 0.5 µm for day 50 and 2 µm for magnified images of day 50.

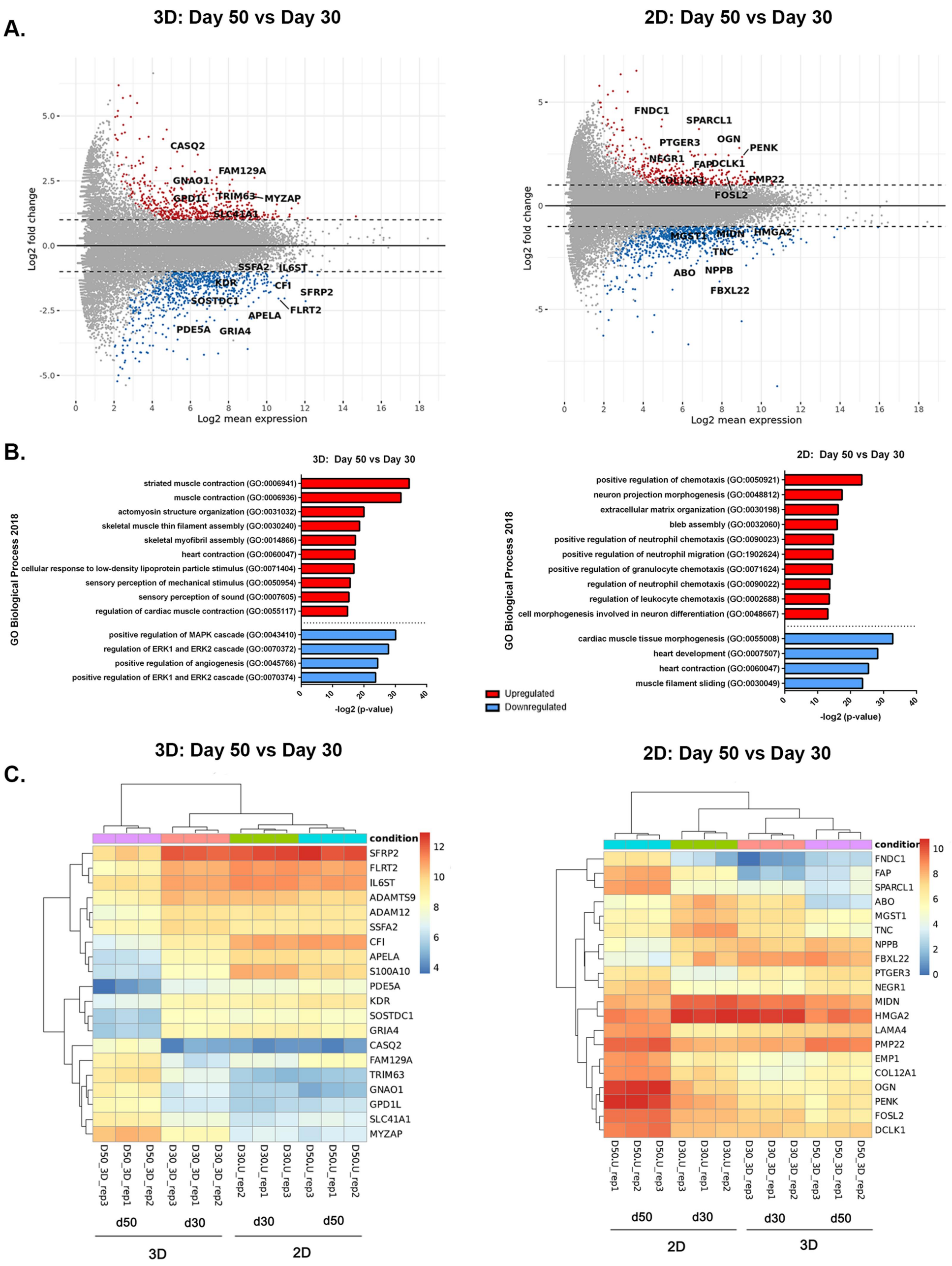

Figure SI-4 – 3D hOCMT cultures induce improved cardiac specification and cardiomyocyte maturation at a transcriptomic level with respect to culture dimensionality and time

A. MA plot of the differentially regulated genes between day 30 and day 50 for 3D hOCMTs (left) and 2D monolayer culture (right) (log2 mean, p.adj <0.05)

B. Graph representing the -log2value adjusted p-value of significantly upregulated (red) or downregulated (blue) GO Biological process categories when comparing between day 30 and day 50 of culture for 3D hOCMTs (left) and 2D monolayer culture (right) (p.adj < 0.05)

C. Heatmap representing the log2fold change for the top 20 differentially regulated genes between day 30 and day 50 of culture for 3D hOCMTs (left) and 2D monolayer culture (right) (p.adj < 0.05)

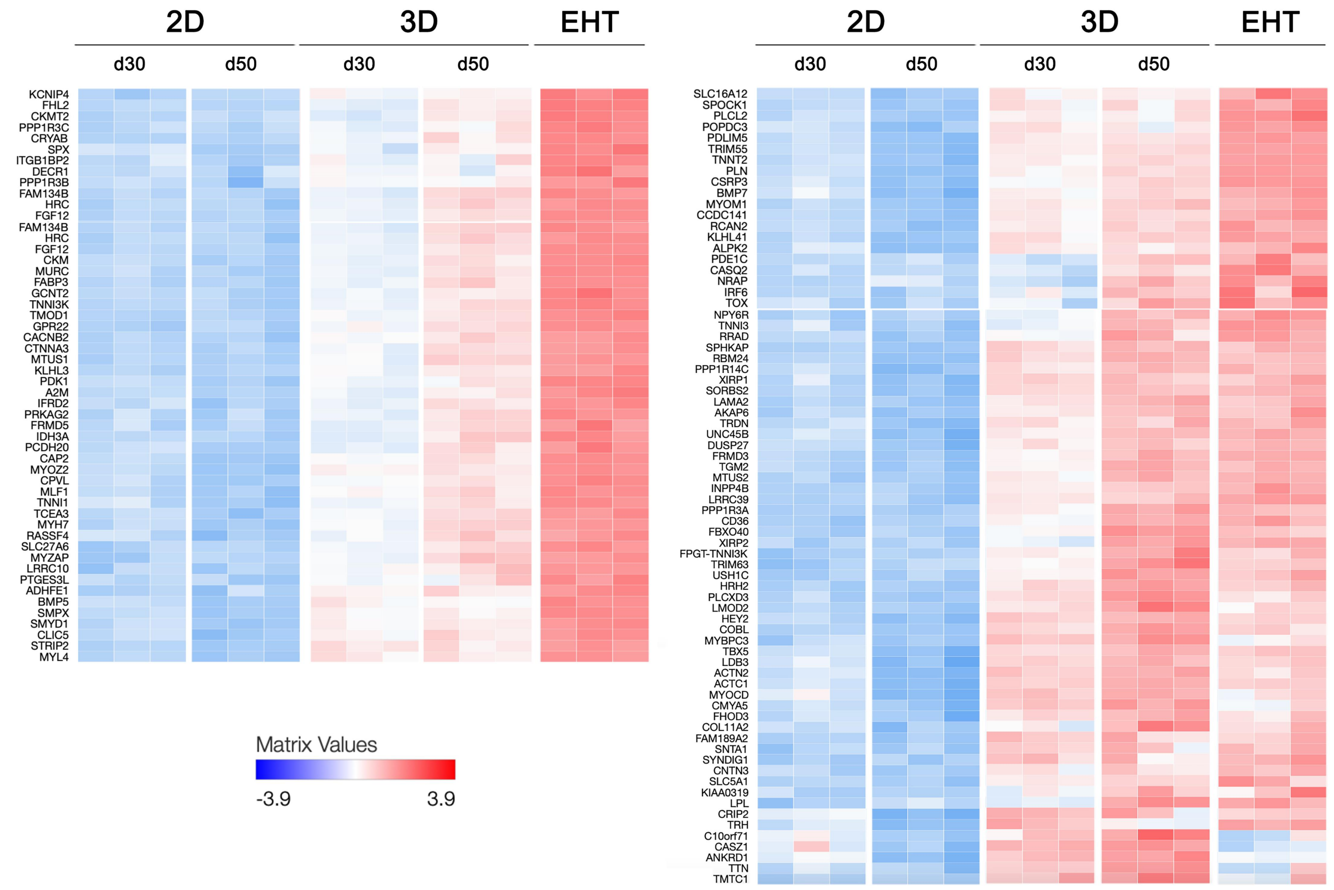

Figure SI-5 – 3D hOCMT cultures show transcriptional similarities to in vitro engineered heart tissues (EHT) with respect to cardiac specification and cardiomyocyte maturation

Heatmap encoding for log2fold changes in selected cardiac genes for the 3D hOCMTs and the 2D monolayers at both time-points in comparison with available datasets for in vitro engineered heart tissues (EHT).

Supplementary Methods

Histology and Immunofluorescence (IF)

After organoid fixation and cryosectioning, to assess the overall histology, the sections were stained with hematoxylin and eosin (H&E) (Sigma Aldrich), and imaged under slide scanner Zeiss Axio Scan Z1 microscope using the bright-field mode.

In addition to 3D hCO cryosections, 2D monolayer cultures were used as controls. As 2D monolayer controls were grown adherently on Matrigel-coated plates, for the IF procedure, the samples were fixed with 4% PFA for 15 minutes, and rest of the IF procedures were performed as previously described in the main text. The resulting samples were stored in PBS with 0.01% Sodium Azide (VWR) at 4°C, and protected from light until confocal image analysis.

See Supplementary table 1 for full list of antibodies used in the study.

RNA-sequencing and Differential Expression analysis (DE analysis)

Sequencing library preparation

500 nanograms of total RNA per sample was used as an input for library preparation using QuantSeq FWD 3'mRNA Library Prep Kit (Lexogen). Briefly, RNA was transcribed into cDNA using oligodT primer (FS1) at half volume compared to the manufacturer's instructions to minimize the off-target products. Following the first strand synthesis, RNA removal and second strand synthesis were performed using the UMI Second Strand Synthesis Mix (USS) containing Unique Molecular Identifiers (UMIs) that allow detection and removal of PCR duplicates. Finally, sequencing libraries were created by PCR with i5 Unique Dual Indexing Add-on Kit for Illumina (Lexogen). The quality and quantity of libraries was determined using Fragment Analyzer by DNF-474 High Sensitivity NGS Fragment Analysis Kit (Agilent Technologies) and QuantiFluor dsDNA System (Promega). Final library pool was sequenced on Illumina NextSeq 500 with 75 bp single-ends, producing about 10 million reads per library.

Data analysis

High-throughput RNA-Seq data were prepared using Lexogen QuantSeq 3' mRNA-Seq Library Prep Kit FWD for Illumina with polyA selection and sequenced on Illumina NextSeq 500 sequencer (run length 1x75 nt). Bcl files were converted to Fastq format using bcl2fastq v. 2.20.0.422 Illumina software for basecalling. 6-nt long UMIs were extracted and subsequently used for deduplication of aligned reads by UMI-tools v. 1.1.1 [1]. As a next step 6-nt long barcode sequence related to Lexogen QuantSeq Library Prep Kit were trimmed using seqtk 1.3-r106 [2]. Quality check of raw single-end fastq reads was carried out by FastQC v0.11.9 [3]. The adapters and quality trimming of raw fastq reads was performed using Trimmomatic v0.36 [4] with settings CROP:250 LEADING:3 TRAILING:3 SLIDINGWINDOW:4:5 MINLEN:35 and adaptor sequence ILLUMINACLIP:AGATCGGAAGAGCACACGTC. Trimmed RNA-Seq reads were mapped against the human genome (hg38) and Ensembl GRCh38 v.94 annotation using STAR v2.7.3a [5] as splice-aware short read aligner and default parameters except --outFilterMismatchNoverLmax 0.1 and --twopassMode Basic. Quality control after alignment concerning the number and percentage of uniquely and multi-mapped reads, rRNA contamination, mapped regions, read coverage distribution, strand specificity, gene biotypes and PCR duplication was performed using several tools namely RSeQC v2.6.2 [6], Picard toolkit v2.18.27 [7] and Qualimap v.2.2.2 [8] and BioBloom tools v 2.3.4-6-g433f [9].

The differential gene expression analysis was calculated based on the gene counts produced using featureCounts tool v1.6.3 [10] with settings -s 2 -T 10 -F GTF -Q 0 -d 1 -D 25000 and using Bioconductor package DESeq2 v1.20.0 [11]. Data generated by DESeq2 with independent filtering were selected for the differential gene expression analysis to avoid potential false positive results. Genes were considered as differentially expressed based on a cut-off of adjusted p-value <= 0.05 and log2(fold-change) ≥1 or ≤-1. Clustered heatmaps were generated from selected top differentially regulated genes using R package pheatmap v1.0.10 [12]. Volcano plots were produced using ggplot v3.3.3 package [13] and MA plots were generated using ggpubr v0.4.0 package [14].

See Supplementary dataset 1 for log2changes and p-values for the different comparisons, and Supplementary dataset 2 for GO BP clustering analysis for the differentially regulated genes at the different comparisons.

IMMUNOFLUORESCENCE				
IF- Primary Antibodies				
**Name**	**Host species**	**Dilution**	**Company**	**Catalog no**
Anti- α-SMA (ACTA2)	Rabbit	1:500	Abcam	ab5694
Anti-ACTN2 (sarcomeric α-actinin)	Mouse	1:800	Sigma Aldrich	A7811
Anti-ASCL2	Sheep	1:50	Thermo Fisher Scientific	PA5-47852
Anti-Brachyury (T)	Goat	1:50	R&D Systems	967332
Anti-CD31 (PECAM-1)	Mouse	1:100	Biolegend	303102
Anti-CX43 (Connexin 43, GJA1)	Rabbit	1:400	Sigma Aldrich	C6219-100UL
Anti-GATA4	Rabbit	1:400	Cell signalling	36966S
Anti-MESP1	Rabbit	1:200	Abcam	ab129387
Anti-MYL2 (α-MLC2, MLC2v)	Rabbit	1:200	Abcam	ab79935
Anti-MYL7 (MLC2a)	Mouse	1:200	Santa Cruz	sc-365255
Anti-NFAT2 (NFATc1)	Rabbit	1:100	Cell Signalling	8032S
Anti-SERCA2 (ATP2A2)	Mouse	1:100	Novus Biologicals	NB300-581
Anti-SM22a (Transgelin, TAGLN)	Rabbit	1:500	Abcam	ab14106
Anti-SOX2	Rabbit	1:400	Cell Signalling	3579
Anti-TE - 7	Mouse	1:100	Sigma Aldrich	CBL271
Anti-TBX18	Rabbit	1:200	Thermo Fisher Scientific	PA5-101921
Anti-TNNT2	Mouse	1:200	Thermo Fisher Scientific	MA5-12960
Anti-TNNT2	Rabbit	1:3000	Sigma Aldrich	HPA015774
Anti-WT1	Rabbit	1:200	Cell Signalling	83535
IF- Secondary Antibodies				
**Name**		**Dilution**	**Company**	**Catalog no**
Alexa Fluor 488 Donkey anti-Rabbit IgG (H+L)		1:500	Thermo Fisher Scientific	A-21206
Alexa Fluor 488 Donkey anti-Mouse IgG (H+L)		1:500	Thermo Fisher Scientific	A-21202
Alexa Fluor 555 Donkey anti-Rabbit IgG (H+L)		1:500	Thermo Fisher Scientific	A-31572
Alexa Fluor 555 Donkey anti- Mouse IgG (H+L)		1:500	Thermo Fisher Scientific	A-31570
Alexa Fluor 546 Donkey anti-Sheep IgG (H+L)		1:500	Thermo Fisher Scientific	A-21098
Alexa Fluor 647 Donkey anti-Goat IgG (H+L)		1:500	Thermo Fisher Scientific	A-21447
Alexa Fluor 647 Goat anti-Mouse IgG (H+L)		1:500	Thermo Fisher Scientific	A-21235
**FLOW CYTOMETRY**				
**Name**		**Dilution**	**Company**	**Catalog no**
Anti Cardiac Troponin T-FITC Clone REA400 (REAfinity™)		1:50	Miltenyi Biotec	130-119-575
Anti-Hu CD90 APC		1:10	Exbio	1A-652-T100
Anti-Hu CD31 BV421		1:10	Biolegend	303123

Supplementary Table 1. List of antibodies used in the study.

List of Supplementary Videos

Supplementary video 1– Representative spontaneously beating long-term 3D hiPSC-derived organotypic cardiac microtissue (hOCMTs) under light microscope (Day 53). Scale bar = 100 µm

Supplementary video 2 – Representative spontaneously beating long-term 3D hOCMT under light microscope (Day 107). Scale bar = 100 µm

Supplementary videos (3-8) – Representative videos of beating long-term hOCMTs before (t=0) & after (t=1) maximum dose of cardioactive drugs on day 50; taken under transmitted light mode of confocal microscope (Scale bars = 200 µm):

3. Control hOCMT (t=0)

4. Control hOCMT (t=1)

5. hOCMT with 0µM isoproterenol (t=0)

6. hOCMT with 1uM isoproterenol (t=1)

7. hOCMT with 0 µM verapamil (t=0)

8. hOCMT with 1 µM verapamil (t=1)

Supplementary videos (9-14) – Representative videos of beating long-term hOCMTs (Day 53) before & after Doxorubicin (Doxo) treatment on day 0 and day 6 of the treatments; taken under transmitted light mode of confocal microscope (Scale bars = 200 µm):

9. Control hOCMT on day 0

10. hOCMT with 0.1µg/mL Doxo on day 0

11. hOCMT with 1µg/mL Doxo on day 0

12. Control hOCMT on day 6

13. hOCMT with 0.1µg/mL Doxo on day 6

14. hOCMT with 1µg/mL Doxo on day 6

List of Supplementary Datasets

Supplementary dataset 1 – log2changes and p-values for the different comparisons

Supplementary dataset 2 – GO BP clustering analysis for the differentially regulated genes at the different comparisons

Supplementary References

[1] T. Smith, A. Heger, and I. Sudbery, "UMI-tools: modeling sequencing errors in Unique Molecular Identifiers to improve quantification accuracy," *Genome Res.*, vol. 27, no. 3, pp. 491–499, Mar. 2017.

[2] H. Li, "seqtk: Toolkit for processing sequences in FASTA/Q formats," *GitHub 767*, 2012. [Online]. Available: https://github.com/lh3/seqtk/. [Accessed: 29-Jan-2022].

[3] S. Andrews, "FastQC A Quality Control tool for High Throughput Sequence Data," *[Online]*, 2010. [Online]. Available: https://www.bioinformatics.babraham.ac.uk/projects/fastqc/. [Accessed: 29-Jan-2022].

[4] A. M. Bolger, M. Lohse, and B. Usadel, "Trimmomatic: a flexible trimmer for Illumina sequence data," *Bioinformatics*, vol. 30, no. 15, p. 2114, Aug. 2014.

[5] A. Dobin *et al.*, "STAR: ultrafast universal RNA-seq aligner," *Bioinformatics*, vol. 29, no. 1, p. 15, Jan. 2013.

[6] L. Wang, S. Wang, and W. Li, "RSeQC: quality control of RNA-seq experiments," *Bioinformatics*, vol. 28, no. 16, pp. 2184–2185, Aug. 2012.

[7] "Picard Toolkit." 2018. Broad Institute, GitHub Repository. 2018. [Online]. Available: https://broadinstitute.github.io/picard/. [Accessed: 29-Jan-2022].

[8] K. Okonechnikov, A. Conesa, and F. García-Alcalde, "Qualimap 2: advanced multi-sample quality control for high-throughput sequencing data," *Bioinformatics*, vol. 32, no. 2, p. 292, Jan. 2016.

[9] J. Chu *et al.*, "BioBloom tools: fast, accurate and memory-efficient host species sequence screening using bloom filters," *Bioinformatics*, vol. 30, no. 23, p. 3402, Dec. 2014.

[10] Y. Liao, G. K. Smyth, and W. Shi, "featureCounts: an efficient general purpose program for assigning sequence reads to genomic features," *Bioinformatics*, vol. 30, no. 7, pp. 923–930, Apr. 2014.

[11] M. I. Love, W. Huber, and S. Anders, "Moderated estimation of fold change and dispersion for RNA-seq data with DESeq2," *Genome Biol.*, vol. 15, no. 12, pp. 1–21, Dec. 2014.

[12] R. Kolde, "Package 'pheatmap,'" *R Package 1.7*, 2015. [Online]. Available: https://cran.r-project.org/web/packages/pheatmap/index.html. [Accessed: 29-Jan-2022].

[13] H. Wickham, "ggplot2," *Wiley Interdiscip. Rev. Comput. Stat.*, vol. 3, no. 2, pp. 180–185, Mar. 2011.

[14] A. Kassambara, "ggpubr:'ggplot2' based publication ready plots," *R package version 0.1 7*, 2018. [Online]. Available: https://rpkgs.datanovia.com/ggpubr/. [Accessed: 29-Jan-2022].
